# Characterization of the Gut-Associated Microbiome in Inflammatory Pouch Complications Following Ileal Pouch-Anal Anastomosis

**DOI:** 10.1371/journal.pone.0066934

**Published:** 2013-09-24

**Authors:** Andrea D. Tyler, Natalie Knox, Boyko Kabakchiev, Raquel Milgrom, Richard Kirsch, Zane Cohen, Robin S. McLeod, David S. Guttman, Denis O. Krause, Mark S. Silverberg

**Affiliations:** 1 Institute of Medical Science, University of Toronto, Toronto, Ontario, Canada; 2 Zane Cohen Centre for Digestive Diseases, Mount Sinai Hospital Inflammatory Bowel Disease Group, Toronto, Ontario, Canada; 3 Department of Animal Science, University of Manitoba, Winnipeg, Manitoba, Canada; 4 Medical Microbiology and Infectious Diseases, University of Manitoba, Winnipeg, Manitoba, Canada; 5 Laboratory Medicine and Pathology, Mount Sinai Hospital, Toronto, Toronto, Ontario, Canada; 6 Department of Cell and Systems Biology, University of Toronto, Toronto, Ontario, Canada; Massachusetts General Hospital, United States of America

## Abstract

**Introduction:**

Inflammatory complications following ileal pouch-anal anastomosis (IPAA) for ulcerative colitis (UC) are common and thought to arise through mechanisms similar to *de* *novo* onset inflammatory bowel disease. The aim of this study was to determine whether specific organisms in the tissue-associated microbiota are associated with inflammatory pouch complications.

**Methods:**

Patients having previously undergone IPAA were recruited from Mount Sinai Hospital. Clinical and demographic information were collected and a pouchoscopy with biopsy of both the pouch and afferent limb was performed. Patients were classified based on post-surgical phenotype into four outcome groups: familial adenomatous polyposis controls (FAP), no pouchitis, pouchitis, and Crohn’s disease-like (CDL). Pyrosequencing of the 16S rRNA V1-V3 hypervariable region, and quantitative PCR for bacteria of interest, were used to identify organisms present in the afferent limb and pouch. Associations with outcomes were evaluated using exact and non-parametric tests of significance.

**Results:**

Analysis at the phylum level indicated that Bacteroidetes were detected significantly less frequently (*P*<0.0001) in the inflammatory outcome groups (pouchitis and CDL) compared to both FAP and no pouchitis. Conversely, Proteobacteria were detected more frequently in the inflammatory groups (*P*=0.01). At the genus level, organisms associated with outcome were detected less frequently among the inflammatory groups compared to those without inflammation. Several of these organisms, including *Bacteroides* (*P<0.0001*)*,* *Parabacteroides* (*P*≤2.2x10^-3^)*,* *Blautia* (*P*≤3.0x10^-3^) and *Sutterella* (*P*≤2.5x10^-3^), were associated with outcome in both the pouch and afferent limb. These associations remained significant even following adjustment for antibiotic use, smoking, country of birth and gender. Individuals with quiescent disease receiving antibiotic therapy displayed similar reductions in these organisms as those with active pouch inflammation.

**Conclusions:**

Specific genera are associated with inflammation of the ileal pouch, with a reduction of typically ubiquitous organisms characterizing the inflammatory phenotypes.

## Introduction

Recent studies have implicated microorganisms in the etiology of several chronic conditions including inflammatory bowel disease (IBD) [1], diabetes [2] and obesity [3]. The evidence for a role of microorganisms in IBD, both Crohn’s disease (CD) and ulcerative colitis (UC), is compelling: numerous polymorphisms in genes associated with innate and adaptive immunity, as well as those associated with barrier function, have been associated with IBD, as have anti-microbial antibodies [4,5,6]. Additionally, some studies have implicated adherent invasive *Escherichia coli* (AIEC) and *Mycobacterium avium* subspecies *paratuberculosis* as potential contributors to pathogenesis [7,8]. On the other hand, decreased frequency of *Faecalibacterium* *prausnitzii* among those with inflammation compared to healthy controls, suggests that this organism may have a protective effect [9,10]. Recent studies have shown that intestinal dysbiosis is associated with disease, and advances in culture-independent sequencing approaches have demonstrated a vast amount of heterogeneity within the microflora of the gastrointestinal tract [11,12]. This highlights the need for further investigation in large and phenotypically diverse cohorts.

Post-surgical models of IBD are useful for studying the role of microbes, as recurrence can be viewed as a surrogate for *de novo* onset of disease. Among UC patients, greater than 20% will require surgical management [13], for which the treatment of choice is a colectomy with ileal-pouch anal anastomosis (IPAA). Colectomy is often considered a definitive treatment for UC, however, *de novo* inflammation of the ileal reservoir (pouchitis) is a common post-surgical complication with prevalence rates ranging from 12% to greater than 50% [14,15]. Additionally, 10-17% of patients go on to develop a CD-like phenotype which is described as the development of abdominal or perianal fistulas or abscesses, or inflammation of the small bowel proximal to the pouch (afferent limb) [16,17]. IPAA is also the treatment of choice among individuals with familial adenomatous polyposis (FAP), however, inflammatory complications of the pouch among this group are very rare. Recent genetic studies have shown that among individuals with IPAA, those with polymorphisms in innate immune and bacterial sensing and recognition genes are at an increased risk for inflammatory complications [18]. However, inflammation rarely develops in the absence of fecal flow, suggesting that this genetic predisposition alone does not itself cause inflammation, and that microbial factors may have a critical role. The aim of this study was to characterize and evaluate the mucosal microbiome of individuals having undergone IPAA for treatment of UC or FAP.

## Materials and Methods

### Ethics Statement

This study was approved by and carried out in accordance with the Research Ethics Board of Mount Sinai Hospital (Toronto, Canada).

### Subject Recruitment

Patients were recruited during regular pouch follow-up at Mount Sinai Hospital (MSH) in Toronto, Canada. Any patients with confirmed UC or FAP and who had undergone IPAA at least one year prior to recruitment were included in the study. Biopsies were taken from within the pouch itself (1 biopsy) and 5-10 cm into the afferent limb (1 biopsy), and were immediately placed into sterile, empty freezer vials and snap frozen in liquid nitrogen. Two additional biopsies from the same locations were sent to the MSH pathology lab for histological scoring. During the pouchoscopy, physicians documented the appearance of the pouch and afferent limb using previously described criteria for pouch inflammation. Peripheral blood was also collected for clinical evaluation of C-reactive protein (CRP) levels.

All subjects were classified into outcome groups based on a combination of long-term complications in conjunction with inflammatory activity at the time of the procedure. Those with FAP were classified as such, while the remaining groups were composed of individuals with UC prior to colectomy. To assess inflammation of both the pouch and afferent limb, endoscopic appearance (erythema, friability, ulceration) and histological (polymorphonuclear leukocyte infiltration, ulceration/erosions) scores at the time of the study endoscopy were considered (Table S1) [19,20]. These traits were utilized as they appear to be objective categories within the PDAI and PAS. Using these categories, a score greater than 3 was applied as the cutoff indicative of inflammation. Individuals who had never been diagnosed with pouchitis and who had no evidence of inflammation of the pouch or afferent limb at the time of sample collection were classified into the “no pouchitis” group; individuals with evidence of inflammation of only the pouch at the time of endoscopy, based on the criteria described above, were classified as “pouchitis”; individuals with documented evidence of inflammation of the afferent limb or proximal small bowel, historically, or at the time of their procedure, or who had developed a stricture or fistula no sooner than one year following the closure of their ileostomy were classified into the “CD-like” group (CDL). This group was made up of individuals who had both active inflammation as well as those without. Individuals with a previous diagnosis of CDL or chronic pouchitis, based on documented evidence of complications and a need for long-term medical therapy, but who had an inflammatory score below our cutoff for both the pouch and afferent limb, and no fistulae, were considered a separate group (“quiescent”) and were not included in the primary analysis.

### Microbial DNA Extraction and Analysis

Biopsies were thawed and processed in two batches at the Department of Animal Science, University of Manitoba (Winnipeg, Canada). The QIAGEN *DNeasy* Blood and Tissue Kit (QIAGEN, California, USA) with an additional bead beating step was used to extract microbial DNA. Barcoded primers specific for the V1-V3 hypervariable region of the 16S rRNA molecule were used for 454 pyrosequencing using the GS FLX Titanium assay (Roche, USA) [21]. Sequences were processed and assigned to a taxonomy using *mothur* (Supplementary Methods).

### Statistical analysis

Taxonomic groups which were detected in less than 5% of the samples were excluded from further analysis. Analyses were performed separately on pouch and afferent limb samples in order to avoid falsely inflating significance. Genus level results were analyzed using presence-absence and frequency data with exact and non-parametric methodologies (Supplementary Methods). Confirmation of results and adjustment for confounding variables was performed using exact logistic regression. FDR corrected p-values below 0.05 were considered significant. All statistical analyses were conducted using STATA version 11.1 (StataCorp, Texas, USA) and R version 2.13.1 (R Foundation for Statistical Computing, Vienna, Austria). Additional analyses were carried out using the Linear Discriminant Analysis (LDA) Effect Size (LEfSe) [22] tool (version 1.1.0).

### Quantitative PCR (qPCR)

Organisms previously implicated in bowel inflammation including *F* *. prausnitzii* , *Clostridial* cluster IV, AIEC, and *Roseburia* were evaluated using qPCR with previously described primer pairs (Table S2). Relative abundance was estimated as has been previously described, using the 16S rRNA gene of the *Eubacteria* as a reference (Supplementary Methods) [23]. Results from individual samples were log_2_ transformed and analyzed using the Kruskal-Wallis test. Additionally, primers specific for the *Bacteroides* genus were used to confirm pyrosequencing results by evaluating the correlation between dichotomized (Matthew’s) and frequency (Spearman) pyrosequencing and qPCR data.

## Results

### Patient phenotypic and clinical characteristics

274 patients were recruited to this study. To ensure a balanced study design, 78 were selected for analysis based on phenotype, with 71 classified into the four primary outcome groups. Seven patients were excluded from the initial analysis having been diagnosed with chronic pouchitis or CDL which was effectively treated with antibiotics or other medications (quiescent). In total, there were 18 patients in the FAP group, 19 no pouchitis, 15 pouchitis, and 19 CDL. Among those classified as CDL, two had only perianal fistulae and an inflammation score of zero in both the pouch and afferent limb (no evidence of inflammation at the time of endoscopy). One additional patient included in this group had evidence of only mild inflammation in both the pouch and afferent limb (inflammatory score less than or equal to 3), with additional features of the CDL phenotype. Inflammatory activity, as measured by our objective inflammatory score, was low among individuals in the FAP and no pouchitis groups, and higher in both the pouchitis and CDL groups in the pouch (P<0.01). In the afferent limb, only individuals in the CDL group displayed evidence of inflammation (P<0.01) (Figure S1).

Interestingly, C-reactive protein (CRP) levels were similarly elevated among each of the UC groups, compared to individuals with FAP (P=0.002) (Table 1). There was an approximately equal split across genders through each of the outcome groups and the mean age at UC or FAP diagnosis and time from ileostomy closure to sampling were approximately the same (Table 1). Smoking was significantly more common (*P*=0.007) among individuals who were in the FAP group. The majority of patients (54%) had taken antibiotics, (ciprofloxacin, metronidazole, or combination) at some point following their pouch procedure (either for pouch complications, or for reasons not related to bowel inflammation). Antibiotic use at the time of pouchoscopy, or during the month immediately prior is documented in Table 1. Individuals in the FAP and no pouchitis groups who were on antibiotics (amoxicillin, cefixime) reported sinus or urinary tract infections as the reason for medication use. One individual in the FAP group took medications because of possible pouchitis-like symptoms. However, endoscopic and histological examinations at several time-points suggest that an inflammatory outcome was not the appropriate diagnosis. Another individual in this group was characterized as having pouch inflammation based on mild pouch erythema and histology showing discrete patchy polymorphonuclear leukocyte infiltration in both the pouch and afferent limb. This individual had no symptoms consistent with pouchitis and was taking Sulindac for periampullary polyps.

**Table 1:** Phenotypic characteristics of individuals among the four outcome groups.

	FAP (n=18)	No Pouchitis (n=19)	Pouchitis (n=15)	Crohn’s disease-like (n=19)	p-value
Gender (% female)	55.6	26.3	26.7	52.6	0.14
Mean age at recruitment (years(SE))	38.1 (2.1)	52.8 (3.3)	41.3 (3.8)	46.3 (3.1)	0.009
Mean age at UC/FAP diagnosis (years (SE))	23.1 (3.0)	31.9 (3.3)	27.8 (3.0)	30.5 (2.9)	0.20
Time from surgery to sample collection (years (SE))	11.1 (1.5)	10.6 (1.7)	9.3 (1.6)	9.9 (1.3)	0.88
C-reactive protein (mean (SE))	2.0 (0.6)	7.1 (3.1)	9.8 (3.4)	7.7 (2.3)	0.002
Antibiotic previous month (% using, SE)	11.1 (7.4)	10.5 (7.0)	13.3 (8.7)	42.1 (11.3)	0.07
Antibiotics ever (% used, SE)	11.1 (7.4)	42.1 (11.3)	73.3 (44.2)	89.5 (30.6)	0.03
Biologics (% using, SE)	0	0	6.7 (6.4)	10.5 (7.0)	0.44
Smoking at recruitment (% using, SE)	41.2 (11.6)	5.3 (16.2)	6.7 (6.4)	5.3 (5.1)	0.007

FAP = familial adenomatous polyposis; SE=standard error. Individuals who were classified as smoking at recruitment reported at least one cigarette per day within the last month.

### Bacterial community diversity and phylum composition of the ileal pouch

The pyrosequencing experiment provided 557,215 (mean of 7143 sequences per sample) raw sequences with a median read length of 453 basepairs (range 250-531). However, following quality trimming and chimera checking, 345,466 (mean of 4429 sequences per sample) high quality reads remained. To ensure that our stringent inclusion criteria did not adversely affect our ability to detect organisms in our samples, Good’s coverage was calculated for each sample, and was on average greater than 92% for all outcome groups. A single sample from the pouchitis group had coverage which was below 80%. Diversity, measured using the Shannon [24] and the inverse Simpson diversity indices [25], was lower among samples obtained from the pouchitis group (Table S3) in the pouch. Results in the afferent limb were similar, although no significant differences between outcome groups were observed at this site.

**Figure 1:**
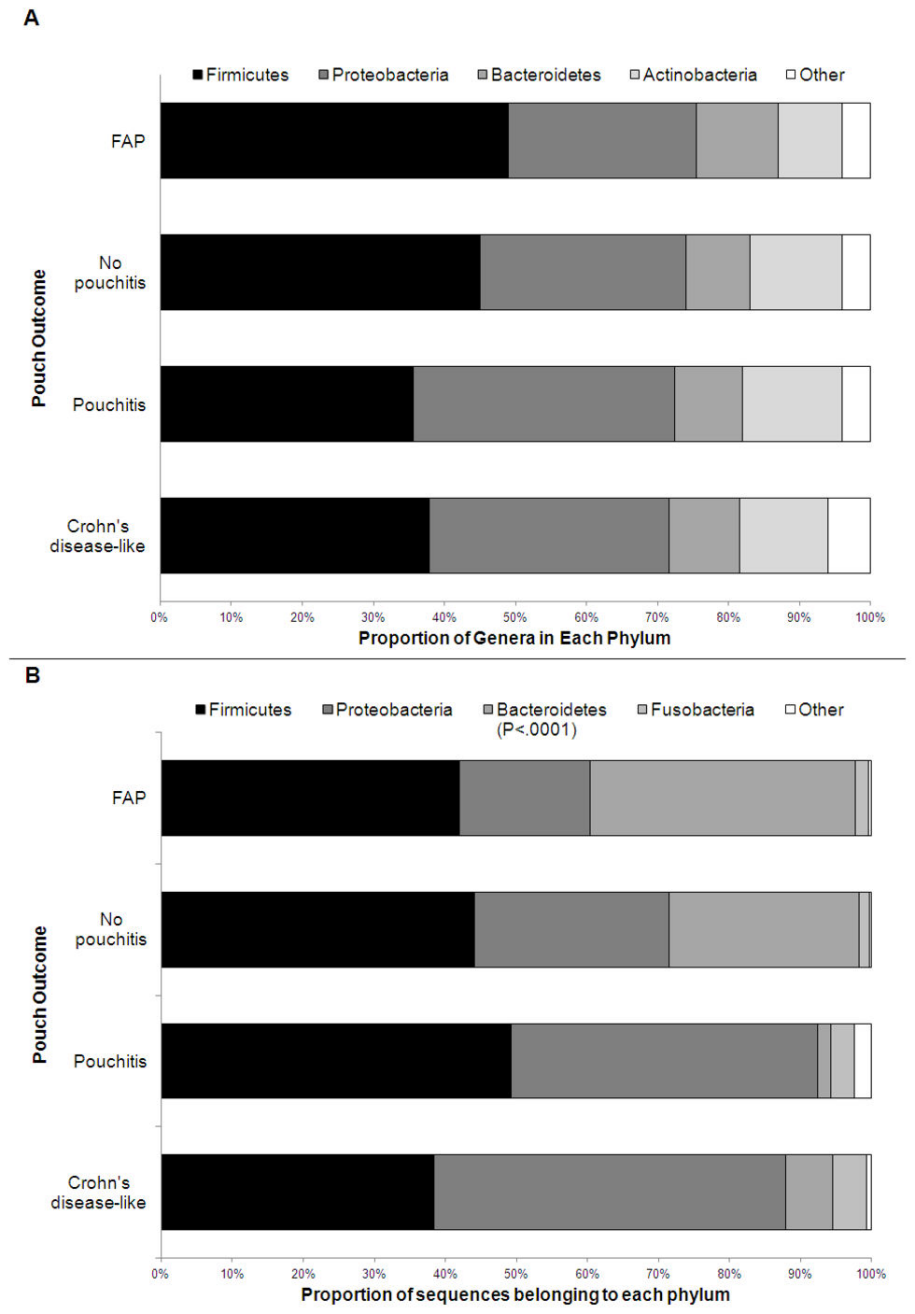
Phylum level comparisons between four outcome groups. A) Proportion of the total number of genera detected in each major phylum. B) Proportion of sequences detected belonging to each phylum. Depicted results are averaged between afferent limb and pouch samples, with significance assessed separately for each site. Individual results for the pouch and afferent limb are included in Figure S2. FAP=familial adenomatous polyposis, CDL=Crohn’s disease-like.

The Firmicutes, Proteobacteria, Bacteroidetes, and Actinobacteria were the most diverse phyla present with the majority of genera detected belonging to one of these groups (Figure 1A; Figure S2). Firmicutes, Proteobacteria, Bacteroidetes and Fusobacteria were numerically the most abundant organisms (Figure 1B; Figure S2). Additionally, Actinobacteria were detected among 72% of the samples, although at very low abundance. There was no significant difference in the number of genera detected per phylum between the four outcome groups. In both the pouch and afferent limb, a significantly greater proportion of sequences in the FAP and no pouchitis groups were Bacteroidetes with, on average, 37.4% and 26.7% of the community composition made up of organisms from these groups compared to the 1.9% and 6.6% in the pouchitis and CDL groups respectively in the pouch (*P*=0.0001) (Figure 1B, Table S4). Results in the afferent limb were similar. While there was no significant association observed in the four-way analysis, Proteobacteria were detected more commonly among individuals with the CDL phenotype compared to FAP (P=0.004). Similar trends were observed between the pouchitis and FAP group, although not reaching statistical significance.

### Microbial composition of the ileal pouch at the genus level

In total, 287 genera were detected. Of these 83 were present in more than 5% of samples. There was no statistical difference in genera positivity or abundance between sampling locations (pouch vs. afferent limb). However, to avoid bias from including multiple samples from the same individual, analysis was conducted separately on the different biopsy sites.

**Figure 2:**
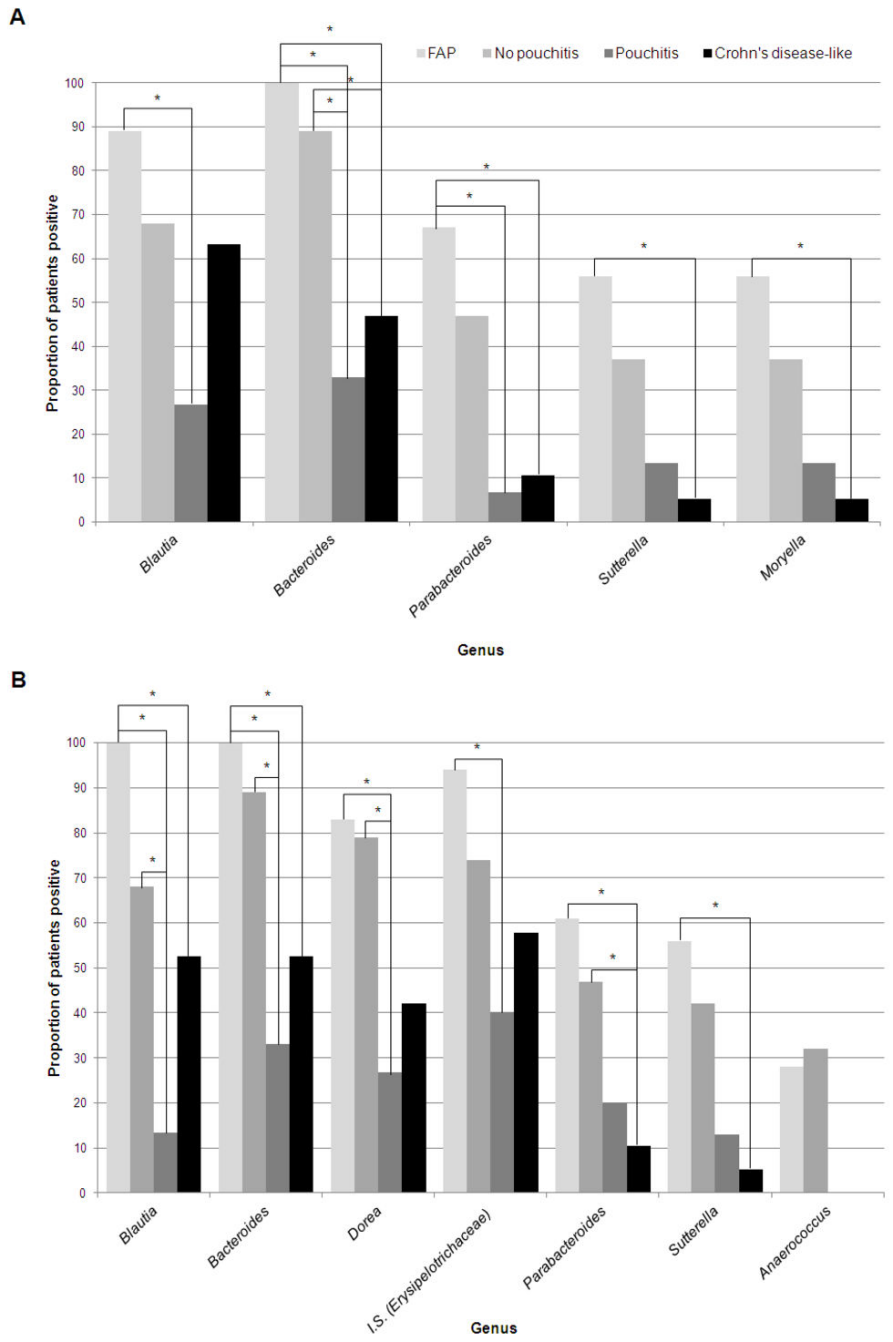
Proportion of patients positive for genera which were significantly associated (P_corr_<0.05) with outcome after correction for multiple testing. * represent pairwise comparisons which were significant after correction. A) Pouch samples. B) Afferent limb samples. I.S.= Incertae sedis.

Fisher’s exact test was used to conduct a four-way comparison evaluating whether different proportions of individuals in each outcome group had a specific genus detected at pouchoscopy. Within the pouch, 15 genera were associated with outcome at a nominal significance threshold of *P*≤0.05, with five ( *Bacteroides* *,* *Parabacteroides* *,* *Moryella* *,* *Sutterella* and *Blautia* ) remaining significant after FDR correction for multiple testing (*P*_*corr*_≤0.05) (Figure 2A, Table S5). In the afferent limb, 16 genera were significantly associated with outcome, with seven ( *Blautia* *,* *Bacteroides* *, Dorea,* *Anaerococcus* *,* *Sutterella* *,* *Parabacteroides* *, and Incertae* Sedis*Erysipelotrichaceae*) remaining significant after correction (Figure 2B; Table S5). These genera were typically detected less frequently among individuals with pouchitis and CDL compared to those with the non-inflammatory outcomes.

**Figure 3:**
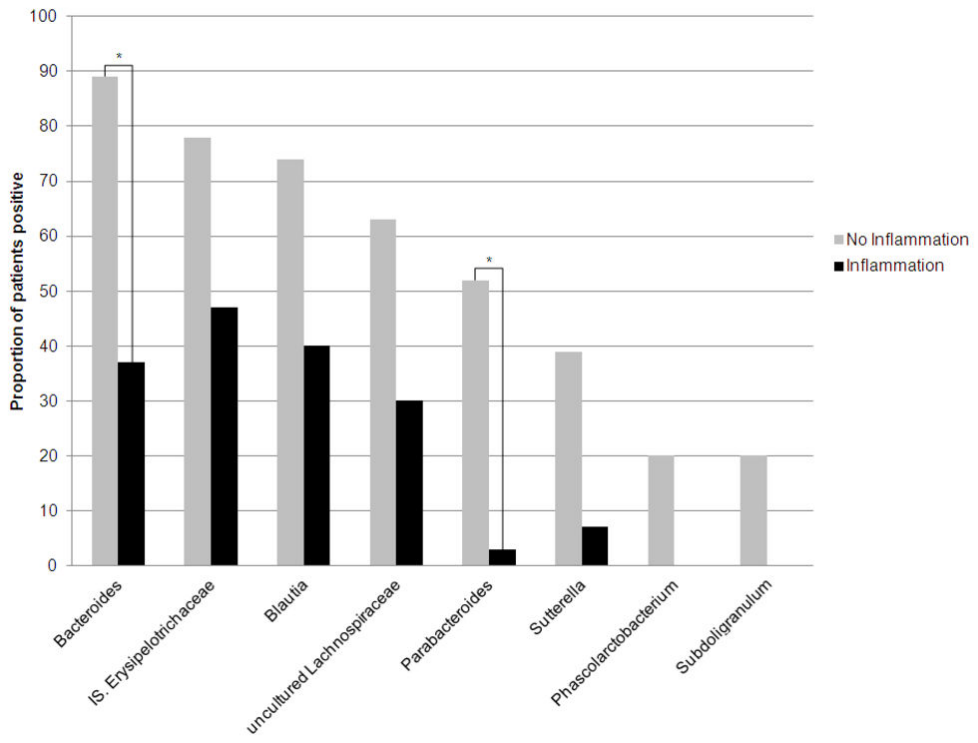
Proportion of patients positive for genera in individuals with inflamed vs not-inflamed pouches. Depicted results are those associated with inflammation at a nominal p-value threshold of P<0.01. * demonstrates associations which remained significant after correction for multiple testing (P_corr_<0.05).

As the CDL group was somewhat heterogeneous, containing individuals both with and without active inflammation at the time of their pouchoscopy, we performed an additional analysis, including only CDL individuals with inflammation at the time of sampling (n=16). The results were similar to those in the previous analysis, although *Moryella* in the pouch and *Anaerococcus* in the afferent limb did not remain significant after multiple testing correction (Figure S3). When we examined the effect of inflammation on bacterial composition we found that genera associated with inflammation were similar to those described in our four group comparison, with *Bacteroides* (*P*=2.5x10^-5^) and *Parabacteroides* (*P*=2.5x10^-4^) less commonly detected among inflamed samples (Figure 3).

We next examined the relationship between quantitative genera abundance and outcome. Results in both locations were similar to those observed for the dichotomized results. In both sites, *Bacteroides* , *Blautia* , and *Sutterella* were positively associated with outcome, as were, *Parabacteroides* and *Moryella* in only the pouch samples (*P*_*corr*_<0.05) (Figure S4). In each case, genera were detected at lower levels in the pouchitis and CDL groups compared to those with non-inflamed tissue.

Across all groups, use of one or more antibiotics at the time of the pouchoscopy or during the preceding month was positively associated with the presence of *Lactobacillus* in both the pouch and afferent limb (pouch *P*=4.6x10^-4^; afferent limb *P*=8.9x10^-4^). Also associated with antibiotic use in the afferent limb were *Dorea* (*P*=4.0x10^-4^) and *Ralstonia* (*P*=1.4x10^-4^), with *Ralstonia* detected more commonly among individuals who were on antibiotics, and *Dorea* less common among individuals on these medications. Several additional genera, including those which were associated with outcome were shown to be marginally effected by antibiotic use at a nominal significance threshold (*P*<0.05), however did not remain significant after correction for multiple testing. Genera abundance data showed similar results, with *Lactobacillus* and *Haemophilus* comprising an increased proportion of the microbiome among individuals on antibiotics in both the pouch and afferent limb, and *Ralstonia* , detected at higher frequency in the afferent limb. *Dorea* was detected at a lower frequency in the afferent limb among individuals on antibiotics. No other medication use appeared to significantly influence microbial composition, although few patients were available for this analysis.

53.5% of our population had taken antibiotics at some point in the past (pre- or post-colectomy) (Table 1). To verify that our observed associations were between inflammatory phenotype and specific genera, and to ensure that our observations were not due to antibiotic use, we performed our analysis again evaluating only individuals who were not on antibiotics during the month (n=57), or 6 months (n=55) preceding their pouchoscopy. Results in the dichotomous analysis for both locations and time points demonstrated that genera previously found to be associated with outcome after correction were all associated in this smaller cohort (*P*<0.05) with the exception of *Moryella* in the pouch and *Parabacteroides* in the afferent limb (Figure S5). All previously described organisms remained significant in the frequency analysis.

Exact logistic regression was performed with smoking, gender and Canadian vs. other country of birth included as co-variates. In both anatomical locations, *Bacteroides* *,* *Sutterella* and *Blautia* , remained significantly associated with outcome (*P*<0.01). *Moryella* , and *Parabacteroides* were associated with outcome only in the pouch and *Dorea* and *Anaerococcus* in the afferent limb (Table 2). Adjusted odds ratios demonstrated a significant decrease in likelihood of pouch inflammatory complications associated with the above genera. No significant results in this pair-wise analysis were detected for the FAP vs. no pouchitis or pouchitis vs. CDL comparisons.

**Table 2:** Association between outcome and bacterial positivity in multivariate analysis with smoking, birth country and gender included as co-variates.

	**FAP vs. Pouchitis**		**FAP vs. CDL**		**No Pouchitis vs. Pouchitis**		**No Pouchitis vs. CDL**	
	**P-value**	**OR**	**P-value**	**OR**	**P-value**	**OR**	**P-value**	**OR**
**Pouch**								
*Bacteroides*	*0.002* *	0.06	*0.002* *	0.04	*0.0005* *	0.04	*0.03*	0.14
*Parabacteroides*	*0.006* *	0.05	*0.004* *	0.05	*0.006* *	0.04	*0.03*	0.13
*Moryella*	*0.04*	0.08	*0.009* *	0.05	*0.30*	0.29	*0.02*	0.07
*Sutterella*	*0.01*	0.05	*0.001* *	0.02	*0.21*	0.26	*0.06*	0.11
*Blautia*	*0.009* *	0.07	*0.31*	0.25	*0.02*	0.11	*1.0*	0.89
**Afferent Limb**								
*Blautia*	*0.0001* *	0.03	*0.02*	0.09	*0.005* *	0.09	*0.50*	0.50
*Bacteroides*	*0.0004* *	0.03	*0.003* *	0.05	*0.003* *	0.08	*0.07*	0.18
*Dorea*	*0.004* *	0.06	*0.003* *	0.05	*0.01*	0.12	*0.11*	0.26
I.S. (*Erysipelotrichaceae*)	*0.04*	0.10	*0.04*	0.08	*0.09*	0.19	*0.54*	0.50
*Sutterella*	*0.06*	0.11	*0.006* *	0.04	*0.14*	0.20	*0.02*	0.07
*Anaerococcus*	*0.34*	0.28	*0.21*	0.22	*0.06*	0.13	*0.008* *	0.06
*Parabacteroides*	*0.13*	0.21	*0.02*	0.10	*0.14*	0.23	*0.03*	0.13

Described nominal p-values marked with a * are those which remain significant after correction for multiple testing (*P*_*corr*_<0.05). Genera tested are those which were significant in the preliminary analysis. No significant associations were seen for the FAP vs No Pouchitis or the Pouchitis vs CDL comparisons. FAP = familial adenomatous polyposis, CDL = Crohn’s disease like. OR = odds ratio I.S.= Incertae sedis

Finally, analysis conducted using LEfSe confirmed many of our previous results, and highlighted additional organisms with potential roles in the inflammatory phenotypes (Figure S6). These included the class Bacilli (phylum Firmicutes) which was detected at higher frequency among inflammatory groups, and Proteobacteria detected more often among individuals with CDL (Figure S6).

### qPCR validation and targeted organism analysis

qPCR was performed on 86 samples from 43 individuals (FAP=12; No Pouchitis=10; Pouchitis=11; CDL=8; quiescent=2). *Bacteroides* qPCR results correlated with those obtained from pyrosequencing (Matthew’s correlation coefficient = 0.78; Spearman correlation coefficient = 0.74). Furthermore, detection rates among the outcome groups were similar with *Bacteroides* detected less frequently in the pouchitis and CDL groups than in either FAP or no pouchitis groups (*P*<0.01). Additional organisms associated with outcome included the *Roseburia* spp. / *E* *. rectale* group (pouch) and *Clostridium* cluster IV (afferent limb). These organisms were present in increased numbers in the FAP group compared to both pouchitis and CDL groups (Figure S7).

### Pyrosequencing results for treated quiescent, medication dependent samples

Seven patients not included in our analysis were classified as quiescent. These individuals were on long term therapy to maintain remission, and displayed no features of inflammation at the time of their study visit. Nominally significant differences (*P*<0.01) were observed between the quiescent and FAP groups in several genera in both the pouch ( *Bacteroides* and *Blautia* ) and the afferent limb ( *Blautia* , *Bacteroides* , and *Incertae* Sedis *Erysipelotrichaceae*) in both the dichotomized and frequency analyses (Figure S8). In both the pouch and afferent limb, there were no significant differences between the pouchitis or CDL groups and the quiescent individuals. Despite the lack of evidence of inflammation, samples taken from individuals on long-term therapy because of a diagnosis of chronic, medication dependent disease, seemed to have a similar microbial profile among pouchitis-associated genera to the inflammatory outcome groups.

## Discussion

These data demonstrate decreased microbial diversity among individuals with pouchitis compared to those with non-inflamed pouches, with broad changes at the phylum and genus level observed. Similar reductions in diversity have also been demonstrated in non-surgical bowel inflammation [1,12]. Interestingly, both the non-parametric Shannon and inverse Simpson indices demonstrated that all three pre-colectomy UC groups had decreased diversity compared to the FAP group. Previous histological evidence suggesting that subclinical inflammation may be present in individuals with pre-colectomy UC, although not in those with FAP may offer an explanation for this finding [26]. Thus, the observation that individuals classified into the no pouchitis group had microbial characteristics intermediate between those seen in individuals with pouchitis and FAP, may be related to subclinical inflammation present in this group which was not captured by our reporting, but which was reflected in increased CRP levels detected in this group. Also of note is the observation that the CDL group did not demonstrate decreased diversity compared to individuals without pouch inflammation. This may be related to the fact that several individuals with CDL were not experiencing active pouch inflammation at sampling, and that the inflammatory activity detected in this group at the time of pouch endoscopy was more heterogeneous than was that observed in the pouchitis group. Indeed, this group had, on average, slightly lower inflammatory scores than did the pouchitis group.

At the phylum level, significantly decreased levels of Bacteroidetes and marginal increases in the Proteobacteria were associated with pouch outcome, among individuals with inflamed pouches compared to those without inflammation. This confirms results from other pouch studies, although results have been mixed in examining these organisms in the context of non-surgical IBD [12,27,28,29,30]. However, our study demonstrates that while Actinobacteria is detected in a majority of samples (72%), it is present at low abundance (mean 0.8%). Fusobacteria, on the other hand, were detected in only 28% of samples, yet among those individuals in whom these organisms were detected they were dominant members of the bacterial community. This contrasts reports from others [27], and can most likely be attributed to the increased sequence coverage, and unique phenotypes explored in our experiment.

Phylum level comparisons, while interesting, are of limited use in predicting biological function. We subsequently demonstrated that specific genera are associated with pouch outcome among our patient cohort. Among the Clostridia group XIVa, *Blautia* *,* *Moryella* and *Dorea* were convincingly associated with outcome, detected more frequently and in greater abundance among individuals without inflammatory complications. Interestingly, a recent study of healthy subjects showed that *Blautia* was one of the only genera detected in samples from all individuals, suggesting that it is a common component of a healthy gut microbiota [31]. Furthermore, previous studies have suggested that individuals with CD, including those in remission, have decreased levels of Clostridia-related organisms detected [32,33]. Both *Bacteroides* and *Parabacteroides* were also strongly associated with outcome. These closely related genera have both been previously implicated in pouch inflammation [27,28]. Decreased levels of *Bacteroides* have also been previously observed in some studies in inflamed UC and CD tissue compared to healthy controls, while other studies have shown increases in this organism to be associated with disease [34,35,36,37]. This further suggests the importance of this organism in bowel inflammation, regardless of host or specific anatomic site. Furthermore, *Bacteroides* species, specifically *B. fragilis*, have been shown to modulate immune function, stimulating immune tolerance through Toll-like receptor (TLR) 2 [38]. *Sutterella* was the only genus from the Proteobacteria phylum associated with outcome, and in contrast with previous studies suggesting a potential pathogenic role for this organism in IBD or no association at all [39], was detected less frequently among inflammatory groups. We noted an increase in the prevalence of Proteobacteria and the class Bacilli among the inflamed groups, however this association was less dramatic. Further study investigating the relevance of these groups of organisms is necessary to determine the significance of this finding.

We specifically evaluated whether organisms previously associated with bowel inflammation, including CD, were also involved in pouch inflammation to determine whether similar biological processes might be of importance in both disorders. Contrary to previous studies, we did not observe an association between *F* *. prausnitzii* or AIEC and inflammatory outcome in our cohort. However, our results do support previous observations that additional members of the Clostridium group [30], and *Roseburia* spp./*E*. *rectale* may be associated with pouch inflammation.

The use of subjects who had an IPAA for FAP, allowed us to evaluate changes occurring in the pouch microbiome which were relevant specifically to individuals with an inherited predisposition to intestinal inflammation. Individuals with FAP rarely develop pouch inflammation, despite the fact that their digestive tract anatomy is identical to those with UC. Additionally, subclinical inflammation may be present in individuals with pre-colectomy UC, without a clinical diagnosis of pouchitis, as our finding of elevated levels of CRP among this group may suggest. This subclinical inflammation has important clinical and experimental implications, and is reflected in our observations illustrating substantial changes between the microbiome of individuals with inflammatory pouch complications compared to those with FAP. More moderate, yet similar alterations occur in the microbiome of individuals with UC when comparing those with and without inflammation. This likely reflects underlying differences in host genetic predisposition in UC patients resulting in specific alterations in the pouch microbiome that characterize the inflammatory phenotypes.

While there were no statistical differences in medication use between outcome groups at the time of or immediately prior to pouchoscopy, it is conceivable that medications, particularly antibiotics, may have impacted the tissue associated microbiota. Yet the described genera were detected significantly less frequently among individuals in the inflammatory outcome groups even when individuals who had taken antibiotics in the preceding month or six months were excluded from the analysis. This would suggest that the effect of inflammation on the microbiome supersedes the impact of antibiotic use. Alternatively, if shifts in the microbiome of the pouch precede and possibly induce pouch inflammation, then the use of antibiotics may not result in long term beneficial effects on bacterial composition or lead to a reversion to a “healthy” pouch microbiome as would be suspected by the typically antibiotic responsive nature of pouchitis. This may in turn be responsible for the often recurrent nature of pouchitis following treatment. It is also possible that past antibiotic use (beyond one month prior to procedure) leads to permanent changes to the microbiome which are detectable long after cessation of medications. Studies evaluating the long-term effect of antibiotics on intestinal microbial composition have shown mixed results with some studies showing a relatively quick reversion to a profile similar to the pre-medication state [40], while others suggest long-term changes, especially with antibiotic use in childhood [41,42]. Interestingly, antibiotic use in childhood has been associated with an increased risk of CD, suggesting that changes to the microbiota which occur early in life, whether they persist long-term or not, may have a drastic impact on subsequent disease susceptibility [43]. However, as the levels of these genera had recovered to detectable levels in FAP and individuals without pouchitis, despite historic antibiotic use (data not shown), yet remained undetectable in most pouchitis and CDL individuals, we conclude that this decrease was associated with the phenotype rather than medication use.

Interestingly, we did not observe differences in the microbial composition of the pouch compared to the afferent limb among patients regardless of the inflammatory status of either site. The anatomical alterations resulting from the IPAA procedure, namely the surgically induced continuity between the pouch and afferent limb, likely promotes similar bacterial community structure between these two locations. Previous studies have documented the occurrence of variability in the microbiome between inflamed and non-inflamed neighbouring sites, although none were consistent across many individuals [29]. This suggests that individual-specific differences in the microbiome may be important in pathology. Further, the absence of evidence for differences between inflamed and uninflamed locations in the same individual suggests that variable host genetics or location-specific gene expression patterns may be of importance in establishing disease location and phenotype. It is also possible that microbial variation results from the inflammatory process itself. In this case, loss of specific organisms may contribute towards propagating disease but not initiate disease processes. Prospective studies are required to evaluate this hypothesis.

Of particular interest, is the observation that the seven individuals who were taking medication for chronically inflamed pouch phenotypes, and who had successfully achieved resolution of their symptoms, did not demonstrate a change in their microbial composition to resemble that of individuals with healthy pouches. While it appears that the samples from individuals with quiescent disease were most similar to those from individuals with pouchitis, it is conceivable that with a larger sample size differences between these groups may have been observed. Additionally, changes at lower taxonomic levels (ie. species), which were undetectable in this experiment could also have been associated with different phenotypes. It is interesting to speculate, however, that perhaps the reason for patients’ continual requirement for medical therapy is their inability to be colonized by the ‘beneficial organisms’ described by our group.

The majority of associations described in this report suggest that a loss of organism diversity is associated with inflammation. It is possible that individuals with pouch inflammation may undergo a loss of specific anti-inflammatory organisms that have important roles in modulating the immune response. Many of the organisms detected less frequently in the inflammatory groups have been previously shown to have anti-inflammatory potential: members of the Clostridia XIVa group ( *Blautia* , *Dorea* and *Moryella* ) produce butyrate, a known anti-inflammatory molecule [44,45], and *Bacteroides* produce vitamin K, which is associated with decreased levels of serum inflammatory markers (ie TNFa, CD40 etc) [46]. Alternatively, the absence of these typically ubiquitous organisms may allow increased colonization of tissue by atypical species. While we did not observe increased prevalence of specific genera in our cohort, it is conceivable that small changes among several groups of organisms may not have been detected. In any event, endogenous gut organisms have the capacity to exert profound effects on the function of the immune system, and play a pivotal role in maintaining gut immune homeostasis [38,47,48]. Such changes in immune function may also be reflected by the presence of anti-microbial antibodies in the serum which are also known to be associated with pouch outcomes [17,49]. In the future, functional metabolites from these organisms may represent novel diagnostic targets or have therapeutic potential for patients with active disease.

Our experiments have demonstrated that a reduction in specific organisms is associated with inflammatory outcomes. These changes are characteristic of both pouchitis and the Crohn’s disease-like phenotype, and include several organisms which have been previously associated with non-surgical IBD. These observations demonstrate that the role of these microbes in bowel inflammation may be common and independent of surgical history or anatomic location of disease. Our previous findings showing that IBD-associated genetic polymorphisms are also important in pouch pathology [18], in conjunction with this work suggests that like non-surgical IBD, pouch inflammation arises in individuals with inherited genetic predisposition and changes in the tissue associated microbiome. However, the event triggering disease onset remains elusive, and will require large, prospective studies to evaluate. 

Sequence accession numbers and availability

Sequences generated in this study are publically available (dbGaP accession number: phs000659.v1.p1).

## Supporting Information

Figure S1 **Distribution of inflammatory activity scores through each of the phenotypic outcome groups.** Mean values for each group are indicated with a red line.
(BMP)

Figure S2 **Phylum level comparisons between four outcome groups for the pouch and afferent limb.** FAP=familial adenomatous polyposis, CDL=Crohn’s disease-like.
(BMP)

Figure S3 **Proportion of patients positive for genera which were significantly associated with outcome (*P*_*corr*_<0.05) following removal of individuals without evidence of inflammation at study pouchoscopy from the CDL group.** * represent pairwise comparisons which were significant (*P*_*corr*_ <0.05) after correction. A) Pouch samples. B) Afferent limb samples. I.S. = Incertae sedis.
(BMP)

Figure S4 **Mean and standard error of the frequency of genera significantly associated with outcome (*P*_*corr*_<0.05) in A) pouch and B) afferent limb.** FAP=familial adenomatous polyposis, CDL=Crohn’s disease-like. Panel i) Bacteroides, ii) other significant organisms.
(BMP)

Figure S5 **Proportion of patients positive for genera which were significantly associated with outcome (*P*_*corr*_<0.05) in the preliminary analysis, among the cohort of patients on no antibiotic therapy (n=57).** * represent pairwise comparisons which were significant (*P*_*corr*_ <0.05) after correction. A) Pouch samples. B) Afferent limb samples. I.S. = Incertae sedis.
(BMP)

Figure S6 **A) Cladograms demonstrating the results obtained through LDA Effect Size (LEfSe) analysis.** Highlighted results are those which were increased in the corresponding group. B) Differential abundance of organisms detected at significantly different frequencies via LEfSe.
(PDF)

Figure S7 **log_2_ transformed relative abundance of organisms of interest from real-time quantitative PCR in the A) pouch and B) afferent limb.** Significant results are marked with an astrix (*P*_*corr*_<0.05). FAP=familial adenomatous polyposis, CDL=Crohn’s disease-like.
(BMP)

Figure S8 **Proportion of individuals in each outcome group positive for genera which were previously associated with pouch inflammatory outcomes.** FAP=familial adenomatous polyposis, Quiescent=seven individuals requiring long-term medical therapy to maintain remission. Lines represent associations between the quiescent group and others which reached nominal significance (P<0.05). A) Pouch samples. B) Afferent limb samples.
(BMP)

Table S1 (PDF)

Table S2 (PDF)

Table S3 (PDF)

Table S4 (PDF)

Table S5 (PDF)
