# Supplementary figures and images for: Characterization of the Gut-Associated Microbiome in Inflammatory Pouch Complications Following Ileal Pouch-Anal Anastomosis
Source: PLoS One. 2013 Sep 24;8(9):e66934. doi: 10.1371/journal.pone.0066934 (PMC3782502; doi:10.1371/journal.pone.0066934)

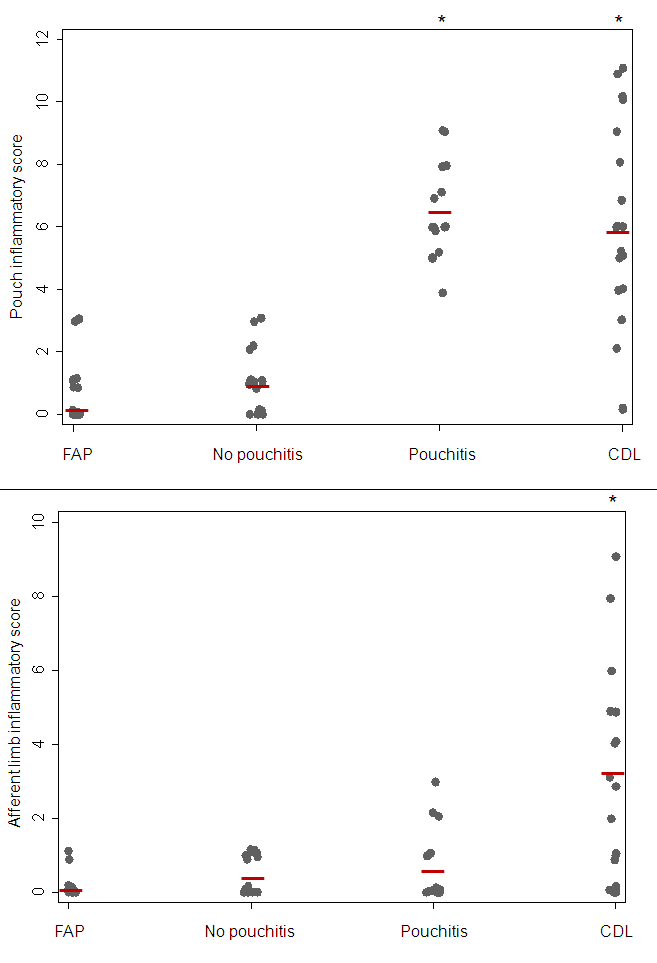

Supplement: Figure S1 — Distribution of inflammatory activity scores through each of the phenotypic outcome groups. Mean values for each group are indicated with a red line. (BMP) [file pone.0066934.s001.bmp]

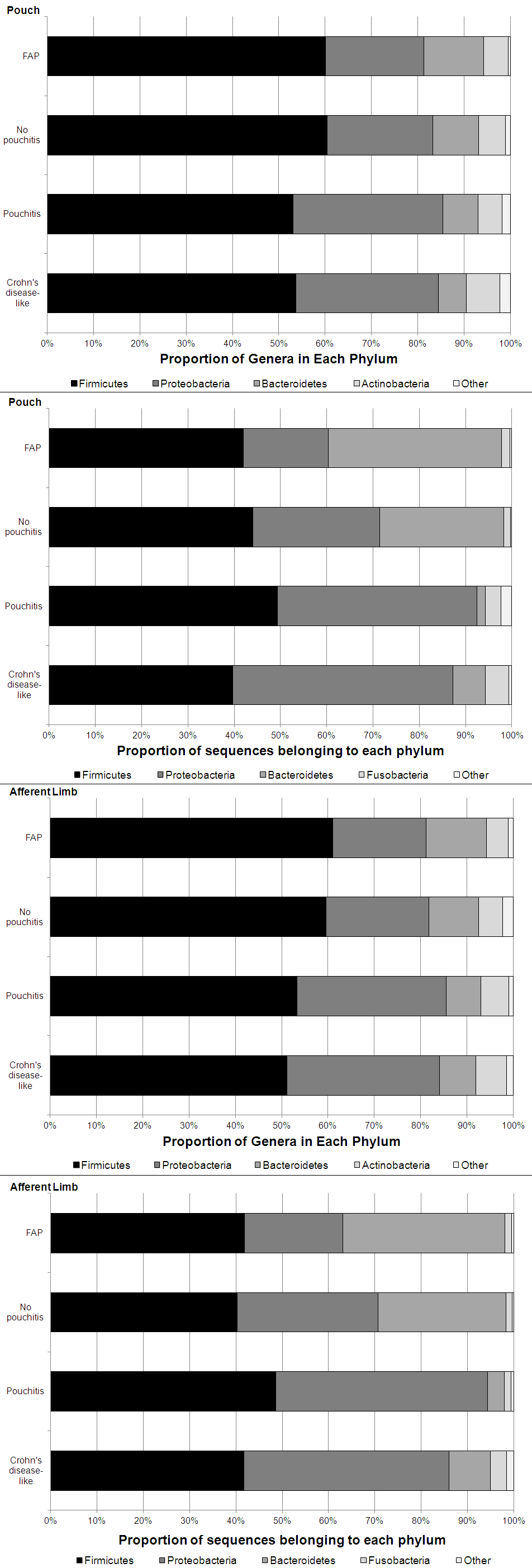

Supplement: Figure S2 — Phylum level comparisons between four outcome groups for the pouch and afferent limb. FAP=familial adenomatous polyposis, CDL=Crohn’s disease-like. (BMP) [file pone.0066934.s002.bmp]

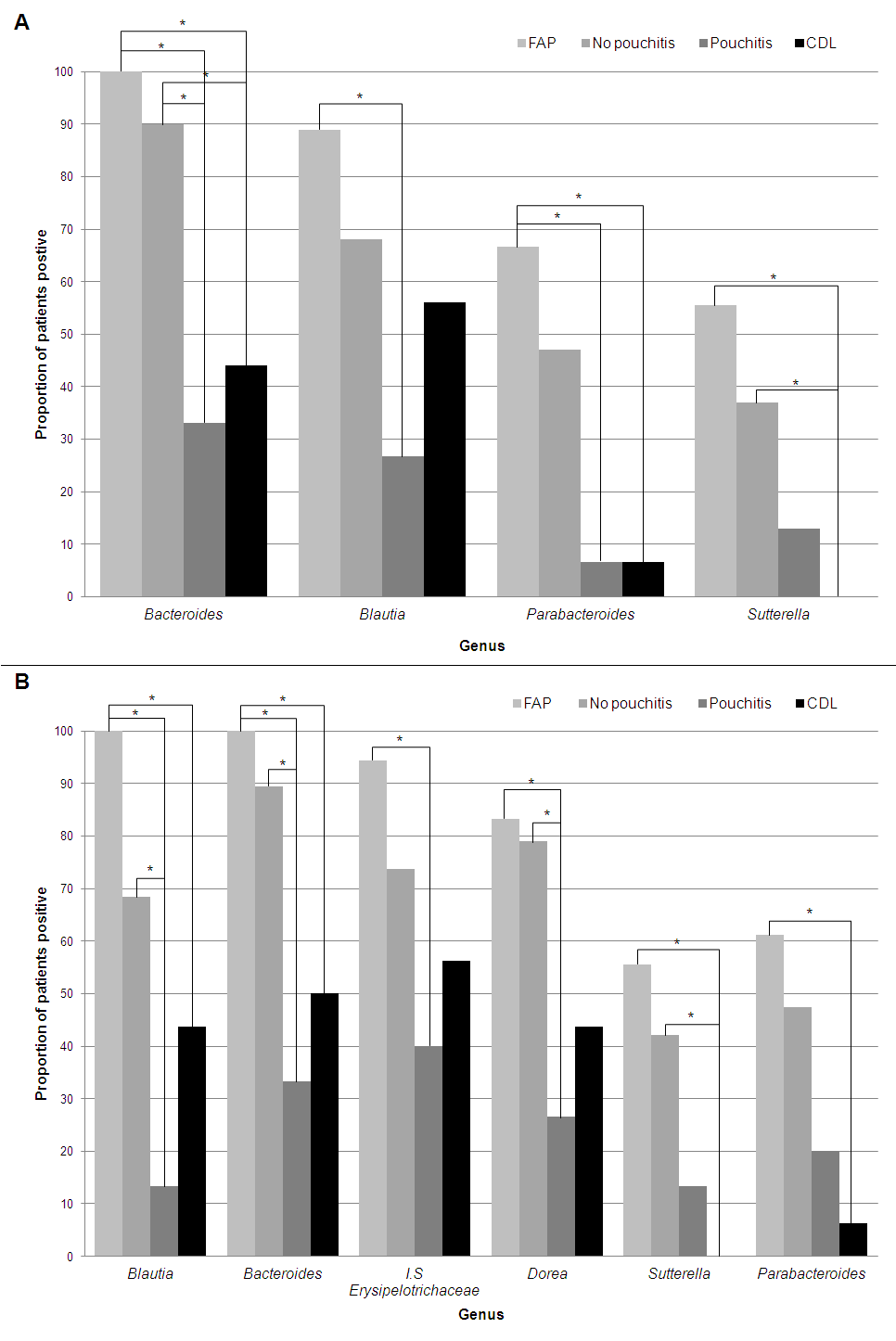

Supplement: Figure S3 — Proportion of patients positive for genera which were significantly associated with outcome (Pcorr<0.05) following removal of individuals without evidence of inflammation at study pouchoscopy from the CDL group. * represent pairwise comparisons which were significant (P corr <0.05) after correction. A) Pouch samples. B) Afferent limb samples. I.S. = Incertae sedis. (BMP) [file pone.0066934.s003.bmp]

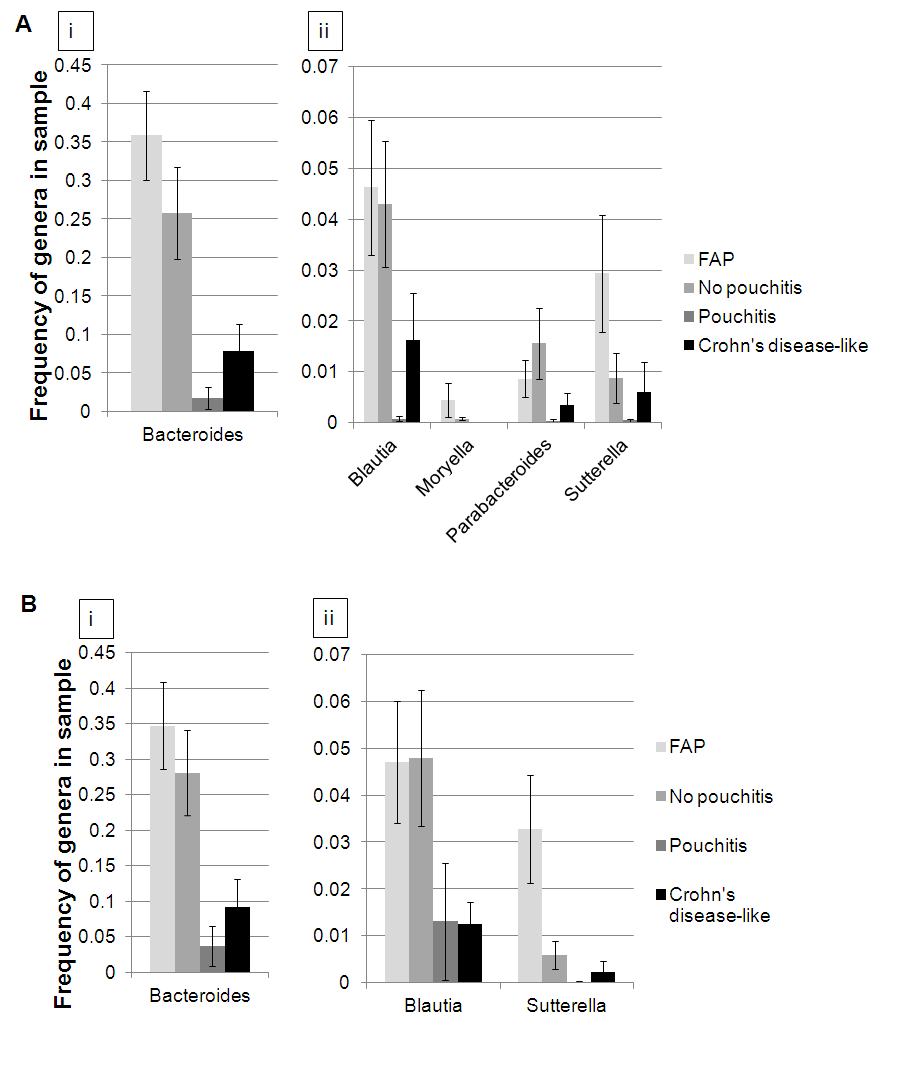

Supplement: Figure S4 — Mean and standard error of the frequency of genera significantly associated with outcome (Pcorr<0.05) in A) pouch and B) afferent limb. FAP=familial adenomatous polyposis, CDL=Crohn’s disease-like. Panel i) Bacteroides, ii) other significant organisms. (BMP) [file pone.0066934.s004.bmp]

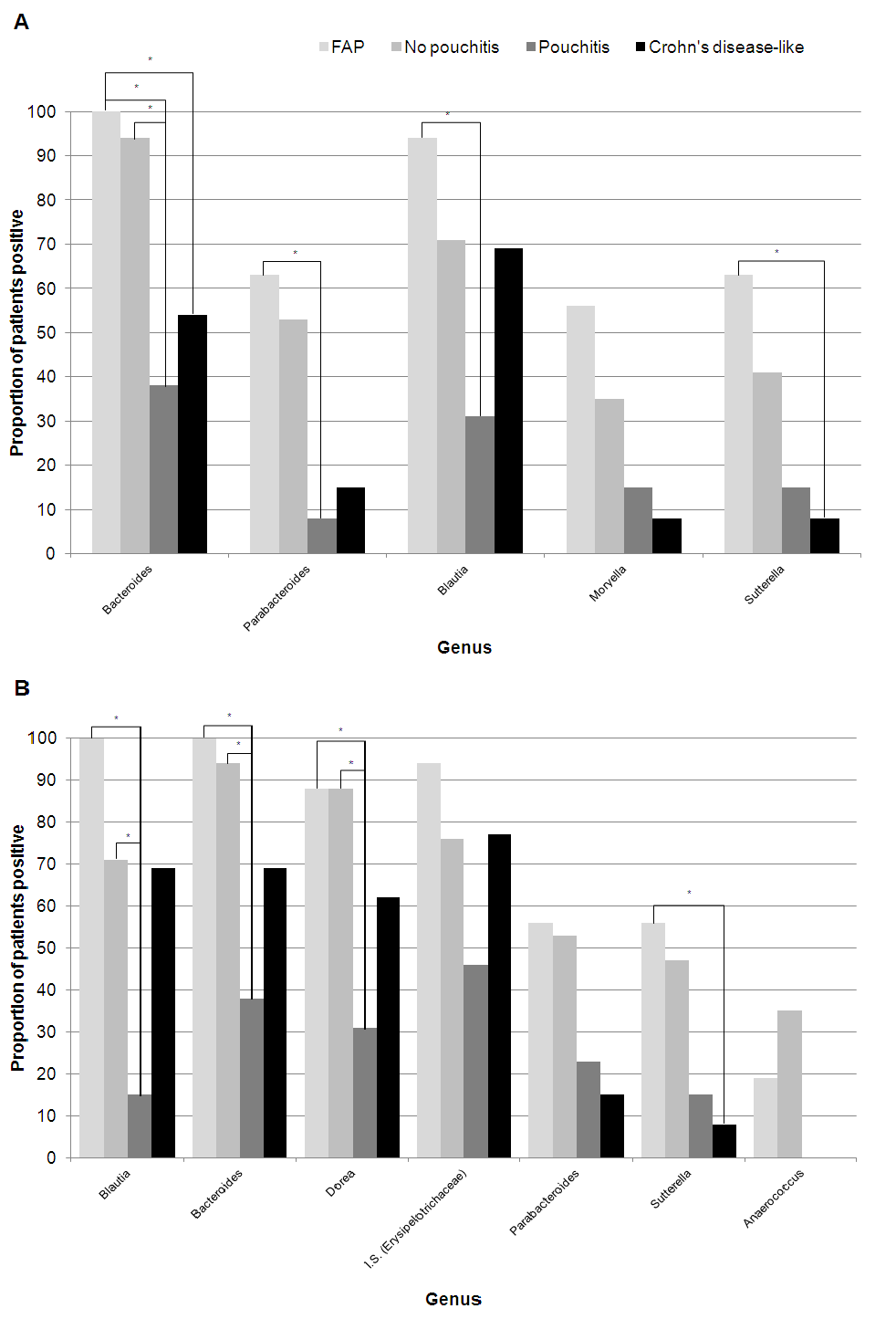

Supplement: Figure S5 — Proportion of patients positive for genera which were significantly associated with outcome (Pcorr<0.05) in the preliminary analysis, among the cohort of patients on no antibiotic therapy (n=57). * represent pairwise comparisons which were significant (P corr <0.05) after correction. A) Pouch samples. B) Afferent limb samples. I.S. = Incertae sedis. (BMP) [file pone.0066934.s005.bmp]

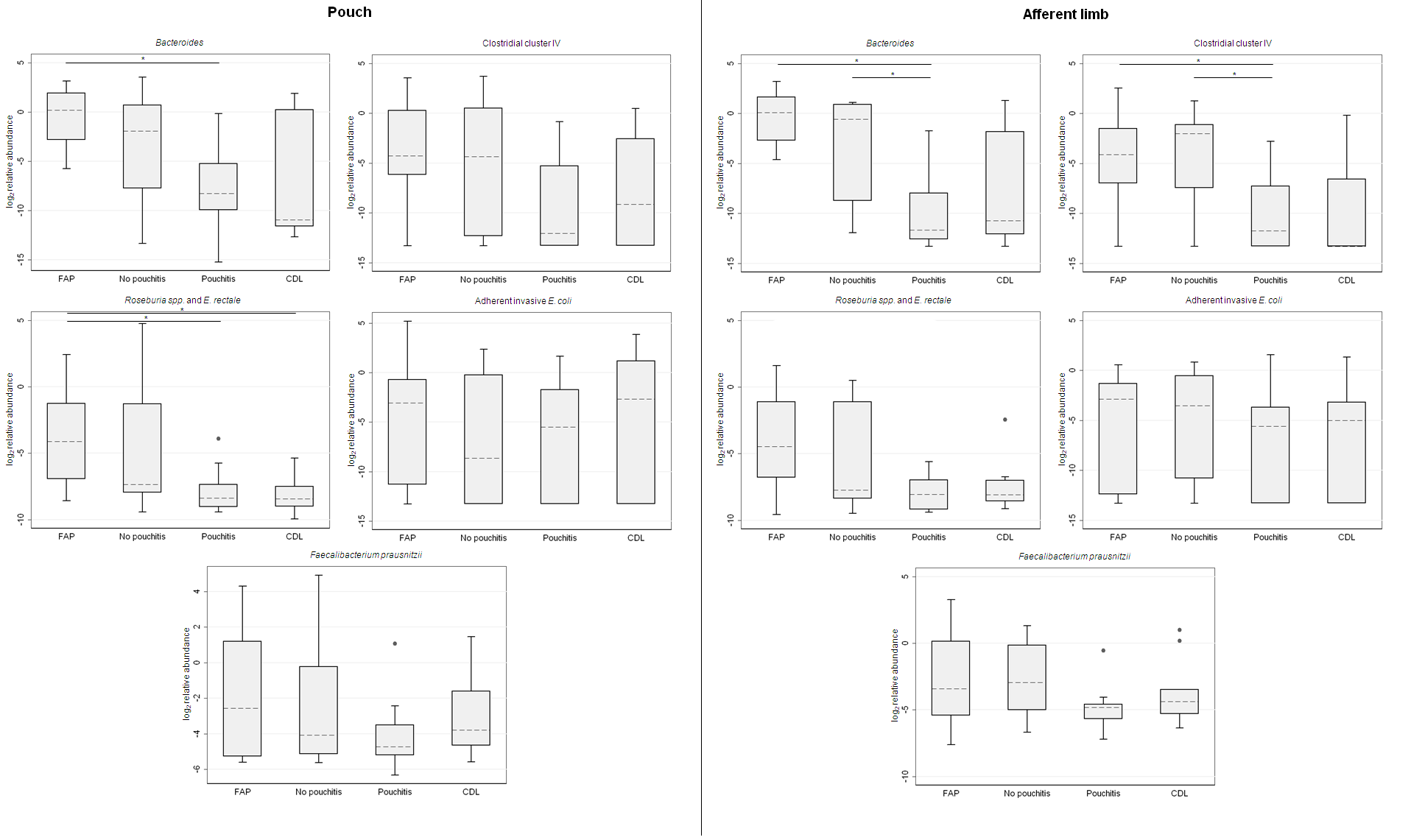

Supplement: Figure S7 — log2 transformed relative abundance of organisms of interest from real-time quantitative PCR in the A) pouch and B) afferent limb. Significant results are marked with an astrix (P corr<0.05). FAP=familial adenomatous polyposis, CDL=Crohn’s disease-like. (BMP) [file pone.0066934.s007.bmp]

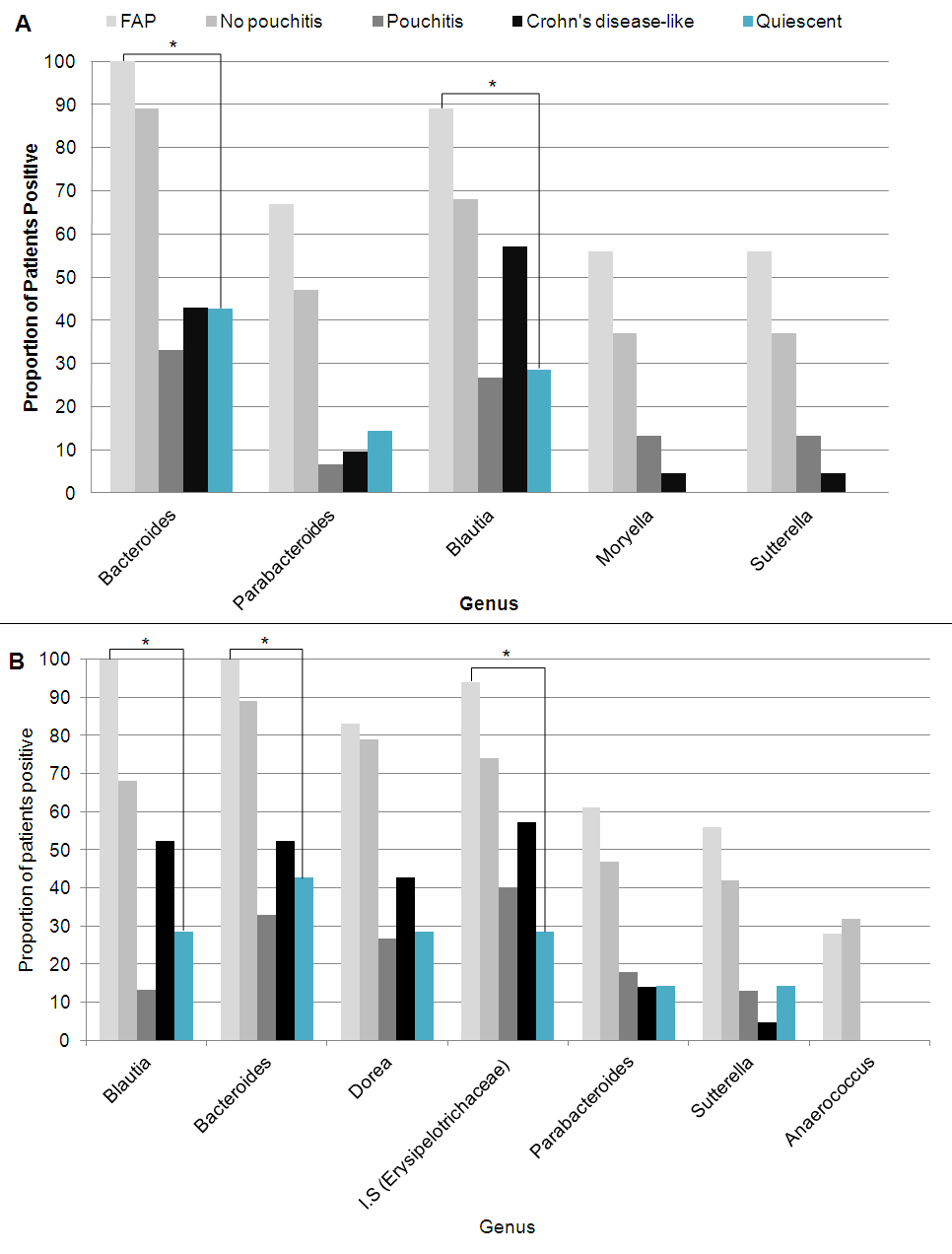

Supplement: Figure S8 — Proportion of individuals in each outcome group positive for genera which were previously associated with pouch inflammatory outcomes. FAP=familial adenomatous polyposis, Quiescent=seven individuals requiring long-term medical therapy to maintain remission. Lines represent associations between the quiescent group and others which reached nominal significance (P<0.05). A) Pouch samples. B) Afferent limb samples. (BMP) [file pone.0066934.s008.bmp]
