# Supplementary material for: Characterization of the Gut-Associated Microbiome in Inflammatory Pouch Complications Following Ileal Pouch-Anal Anastomosis
Source: PLoS One. 2013 Sep 24;8(9):e66934. doi: 10.1371/journal.pone.0066934 (PMC3782502; doi:10.1371/journal.pone.0066934)
Supplement: Figure S6 — A) Cladograms demonstrating the results obtained through LDA Effect Size (LEfSe) analysis. Highlighted results are those which were increased in the corresponding group. B) Differential abundance of organisms detected at significantly different frequencies via LEfSe. (PDF) [file pone.0066934.s006.pdf]

Cladogram - Pouch samples, four group comparison

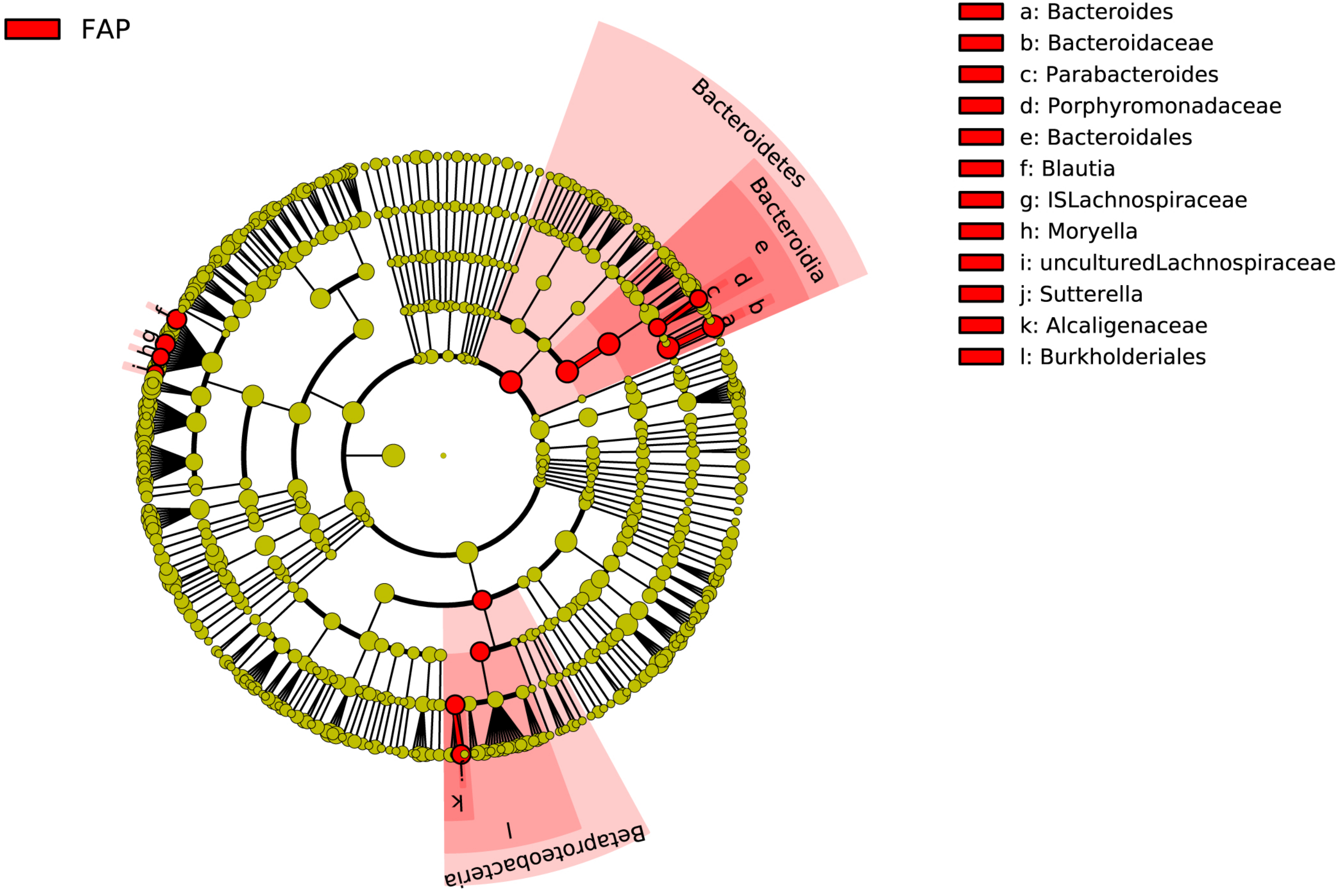


Significant associations as highlighted, with outcome group(s) where increased proportion was detectable indicated

Cladogram - Pouch samples, FAP vs Pouchitis

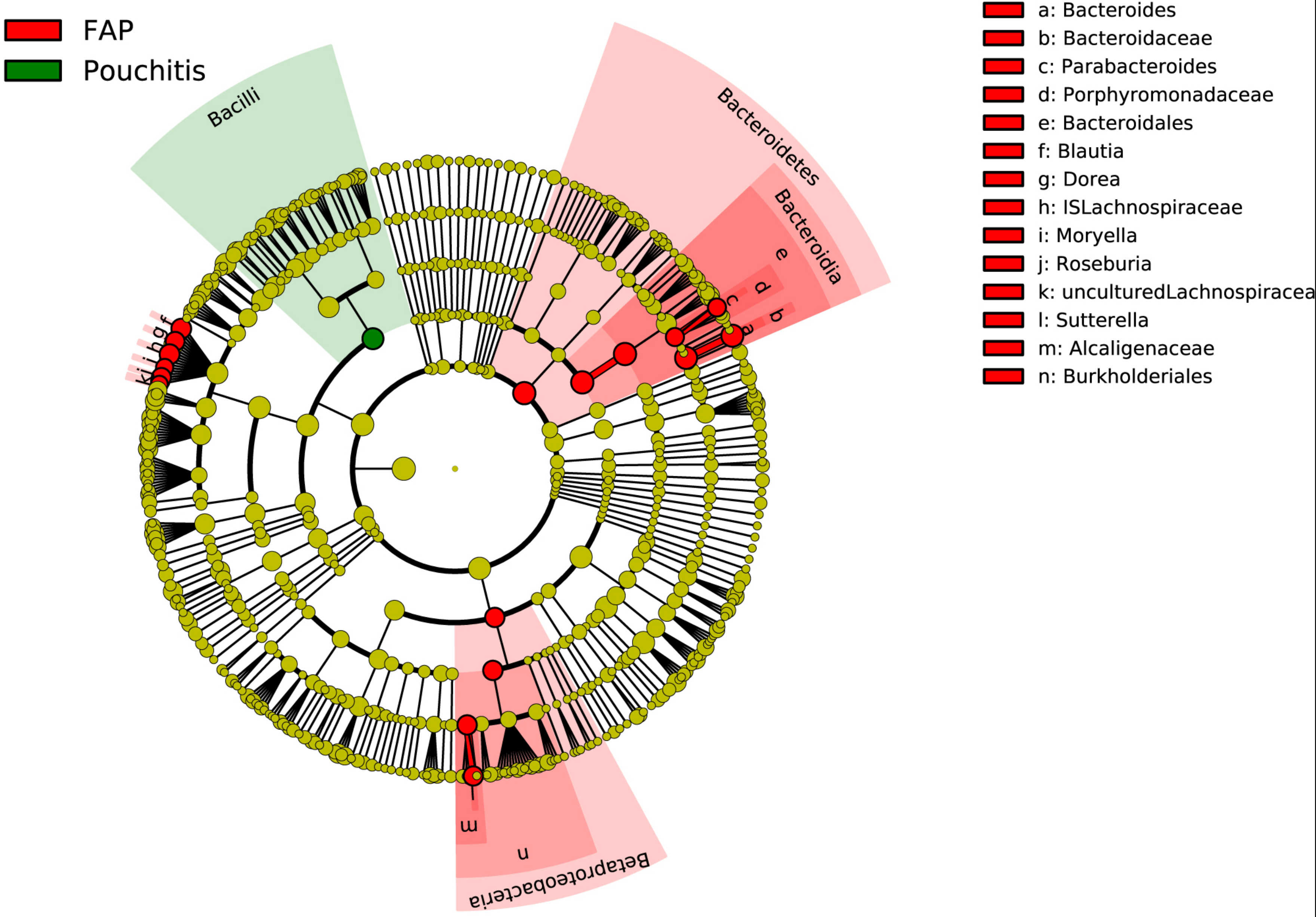


Significant associations as highlighted, with outcome group(s) where increased proportion was detectable indicated

Cladogram - Pouch samples, FAP vs Crohn's disease-like

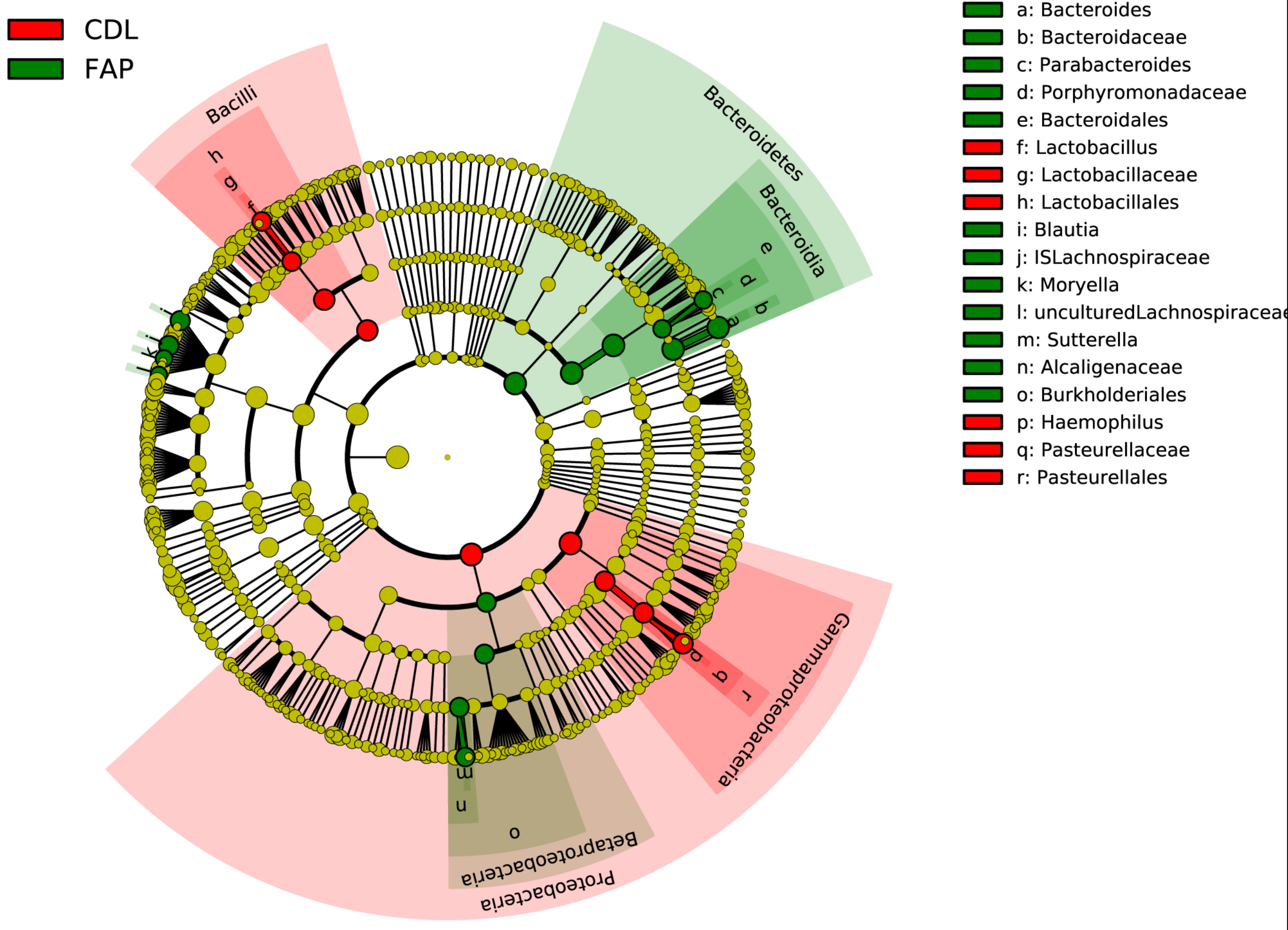


Significant associations as highlighted, with outcome group(s) where increased proportion was detectable indicated

Cladogram - Pouch samples, No Pouchitis vs Crohn's disease-like

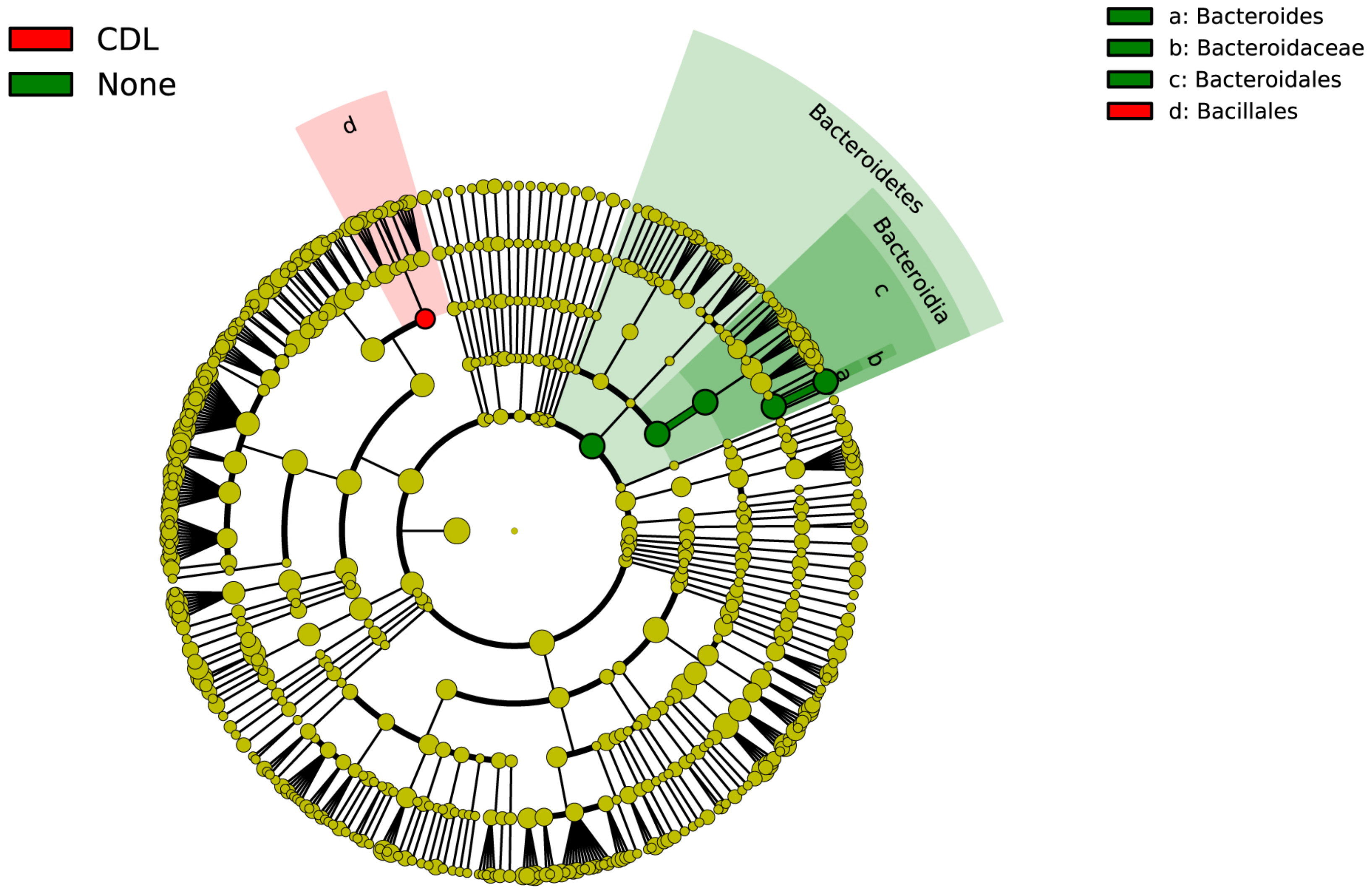


Significant associations as highlighted, with outcome group(s) where increased proportion was detectable indicated

Cladogram - Pouch samples, No Pouchitis vs Pouchitis

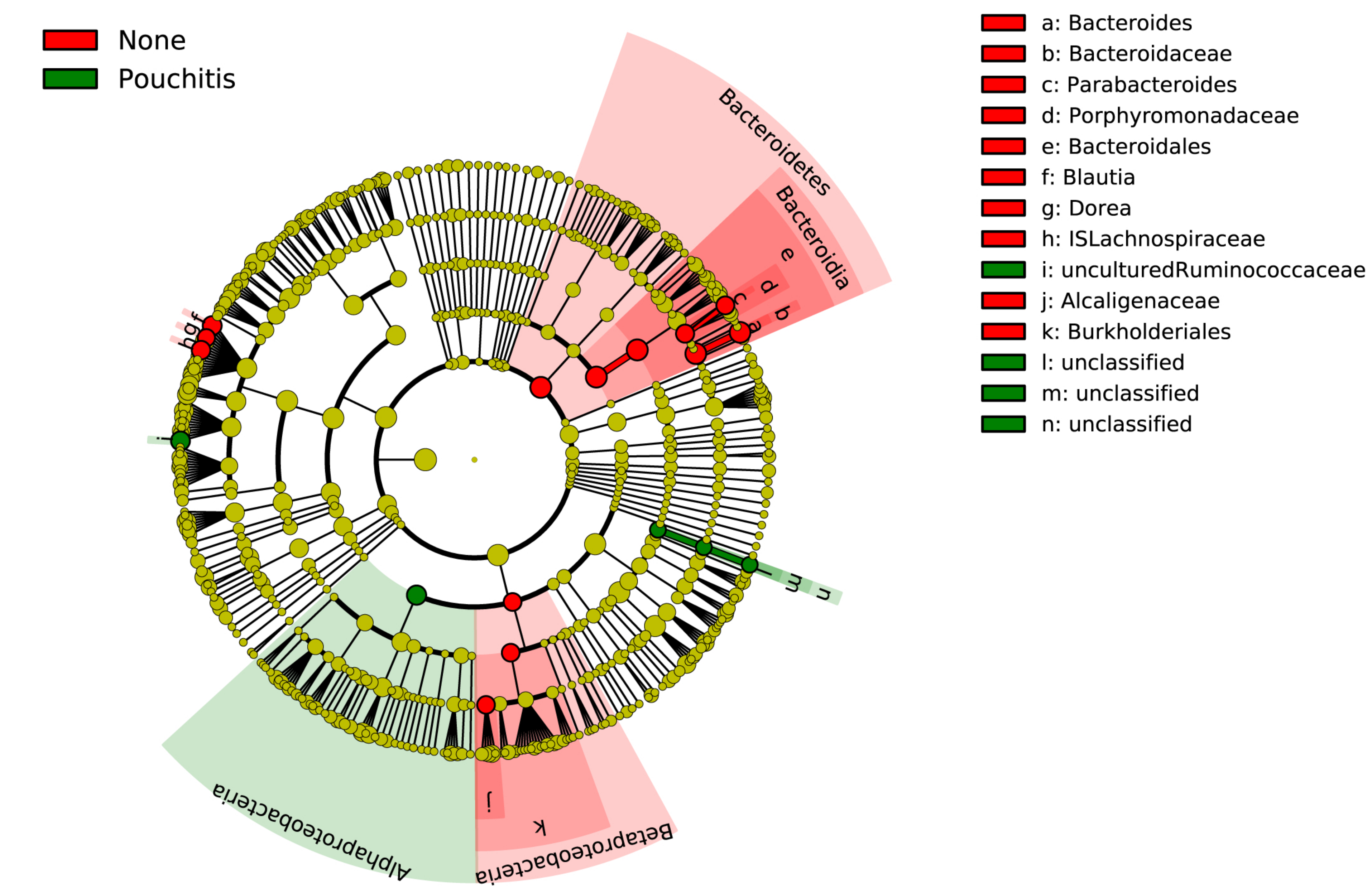


Significant associations as highlighted, with outcome group(s) where increased proportion was detectable indicated

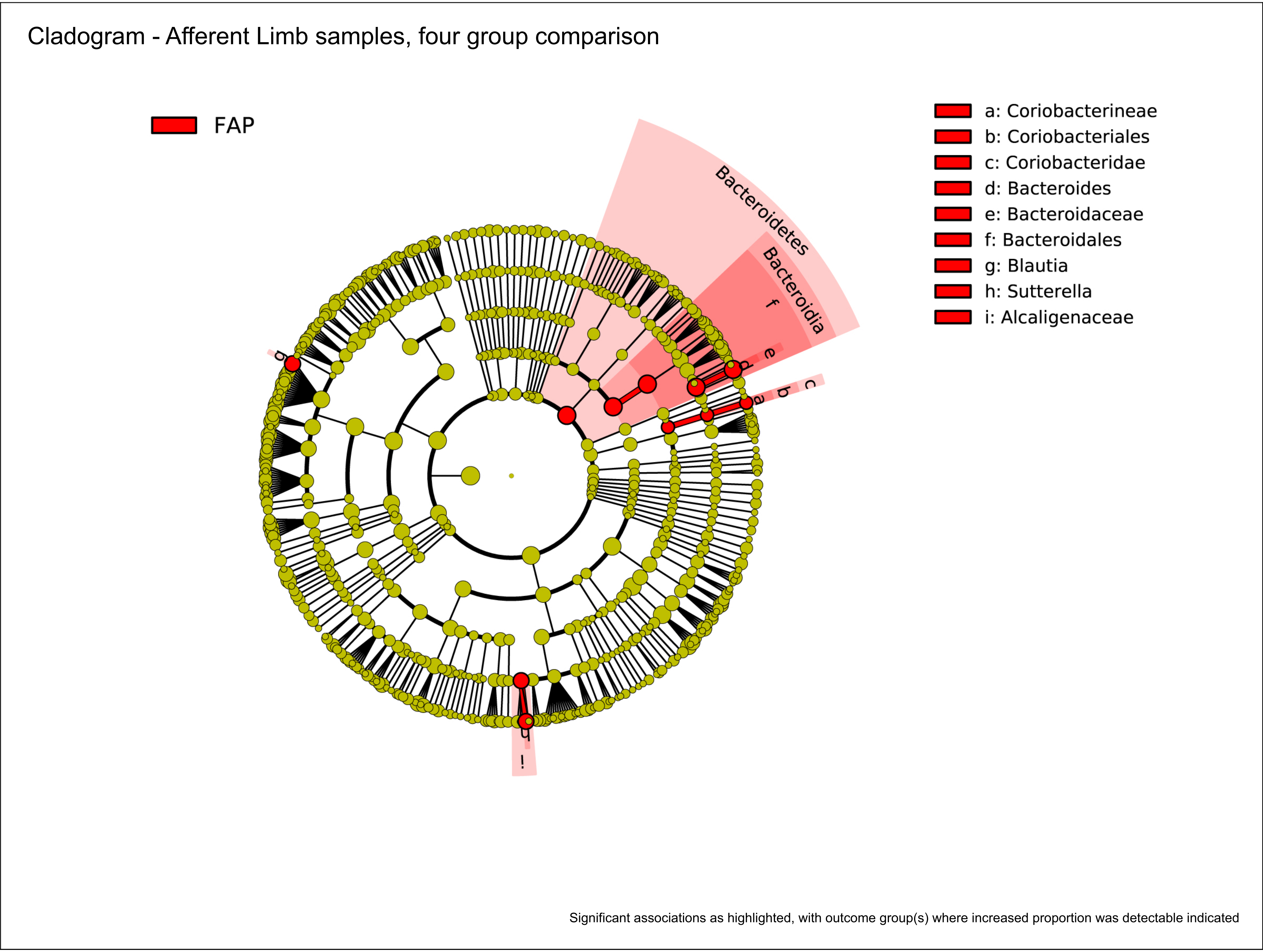
Cladogram - Afferent Limb samples, four group comparison
FAP
a: Coriobacterineae
b: Coriobacteriales
c: Coriobacteridae
d: Bacteroides
e: Bacteroidaceae
f: Bacteroidales
g: Blautia
h: Sutterella
i: Alcaligenaceae
Bacteroidetes
Bacteroidia
Significant associations as highlighted, with outcome group(s) where increased proportion was detectable indicated

Cladogram - Afferent Limb samples, FAP vs Pouchitis

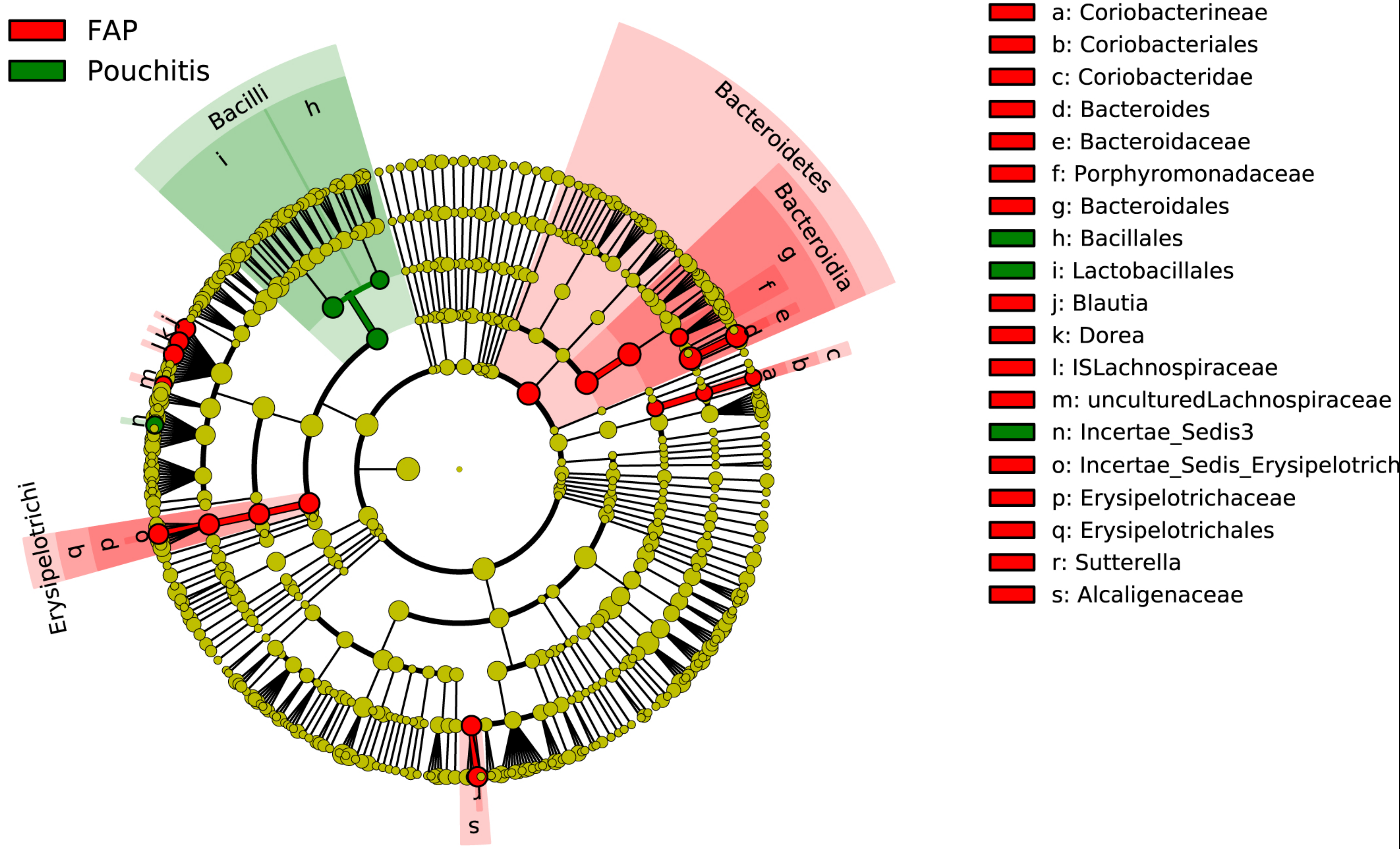


Significant associations as highlighted, with outcome group(s) where increased proportion was detectable indicated

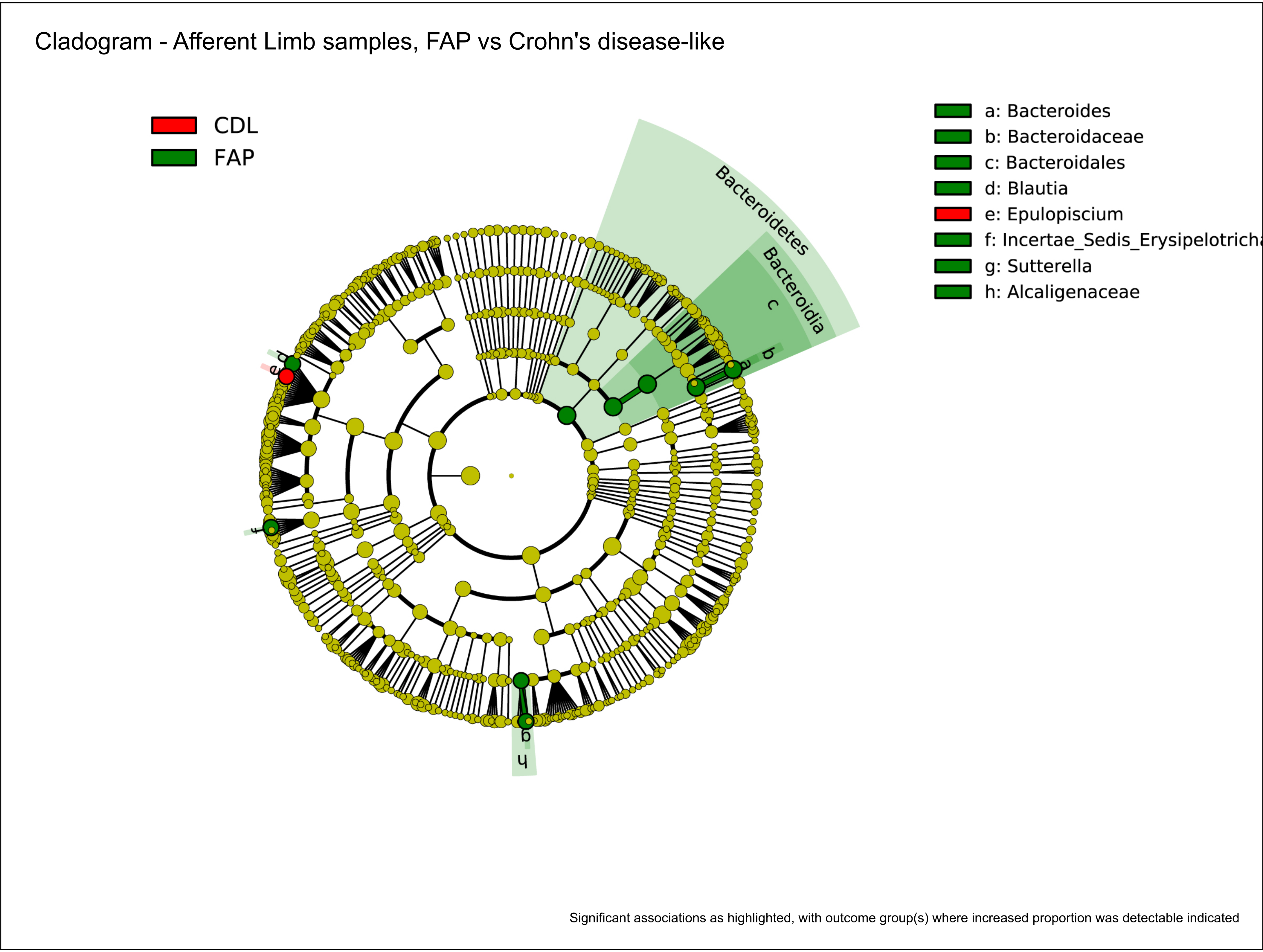
Cladogram - Afferent Limb samples, FAP vs Crohn's disease-like
CDL
FAP
a: Bacteroides
b: Bacteroidaceae
c: Bacteroidales
d: Blautia
e: Epulopiscium
f: Incertae_Sedis_Erysipelotricha
g: Sutterella
h: Alcaligenaceae
Bacteroidetes
Bacteroidia
Significant associations as highlighted, with outcome group(s) where increased proportion was detectable indicated

Cladogram - Afferent Limb samples, No Pouchitis vs Pouchitis

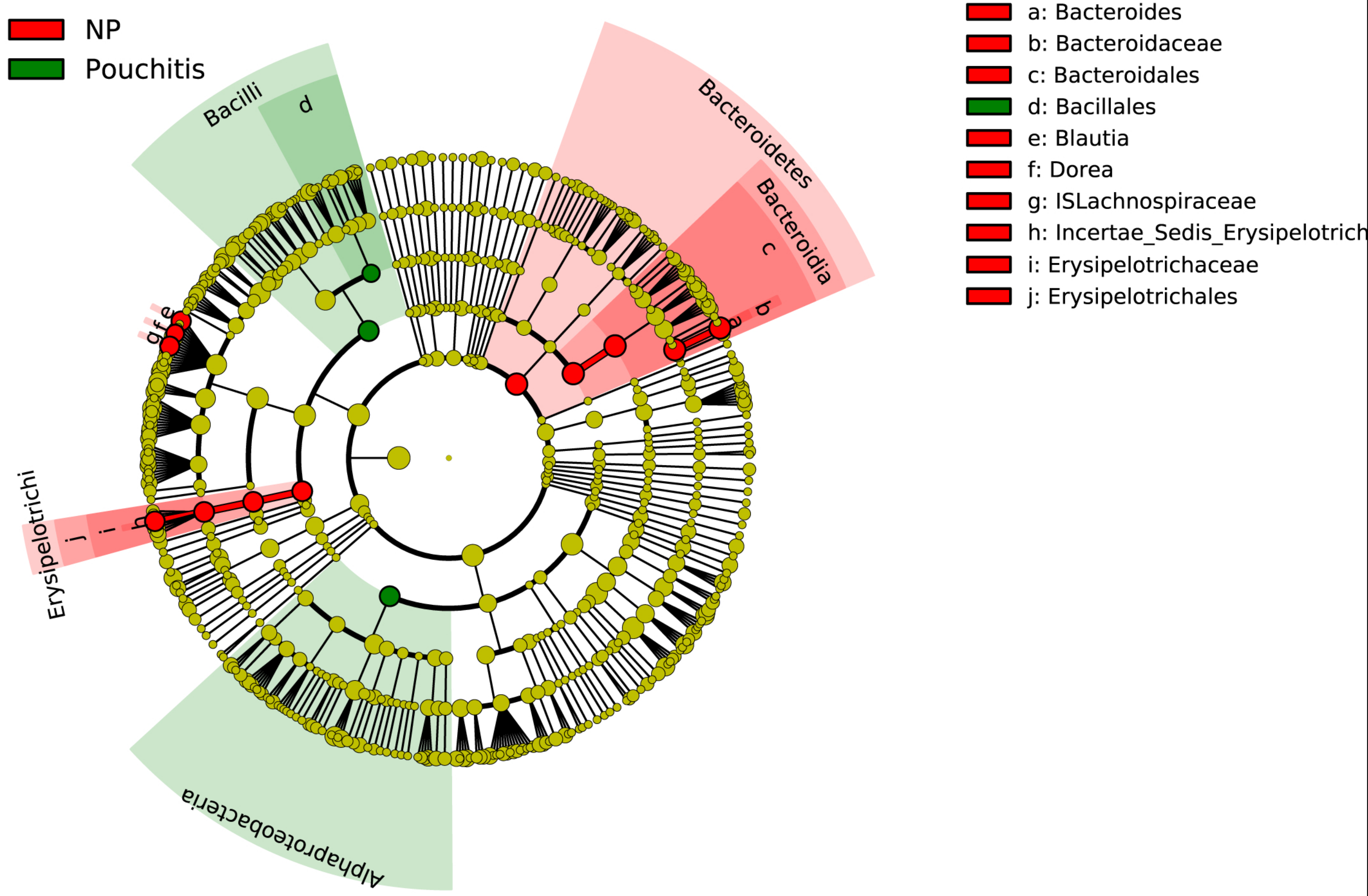


Significant associations as highlighted, with outcome group(s) where increased proportion was detectable indicated

Cladogram - Afferent Limb samples, No Pouchitis vs Crohn's disease-like

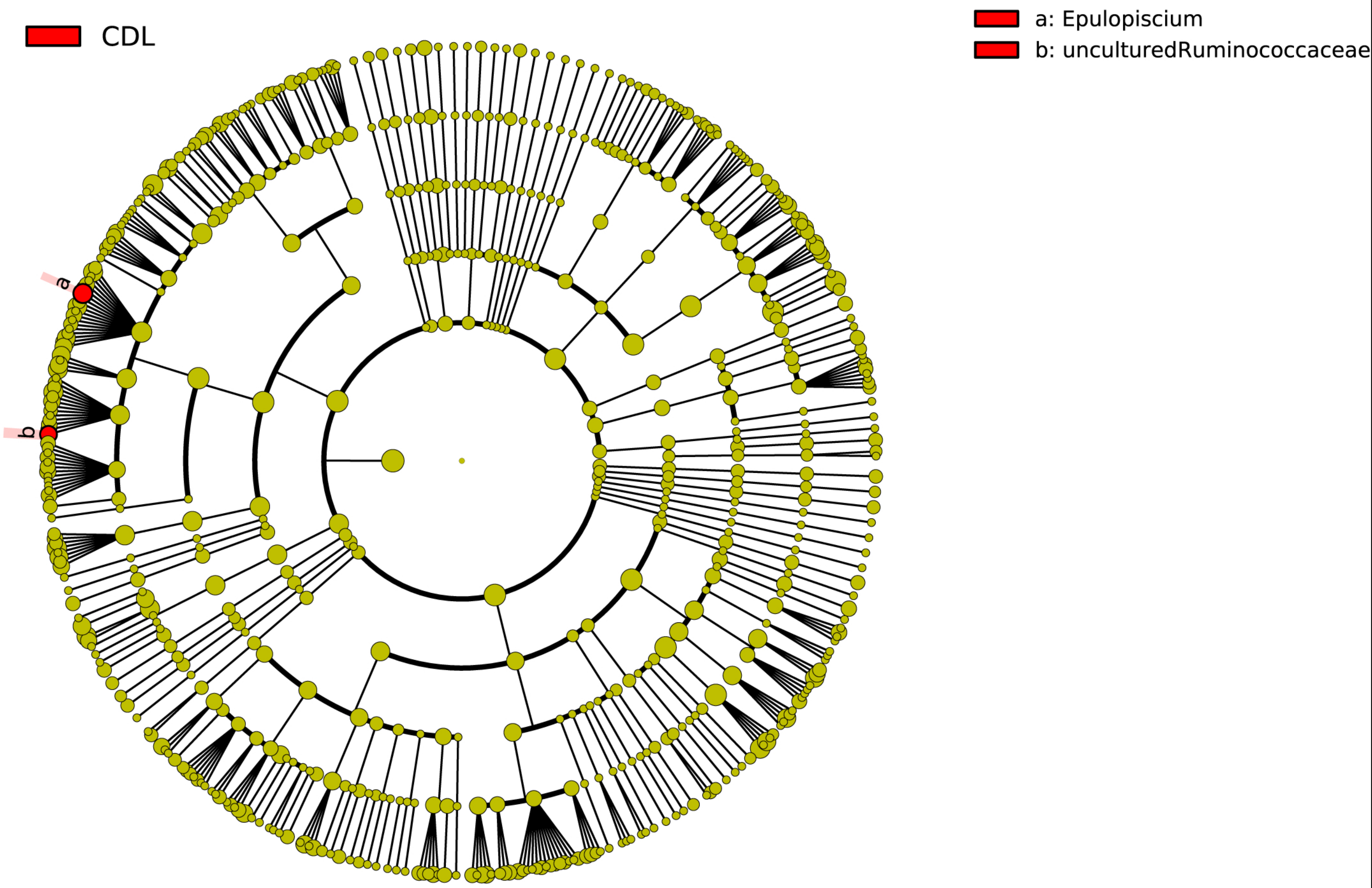


Significant associations as highlighted, with outcome group(s) where increased proportion was detectable indicated
