# Supplementary material for: Characterization of the Gut-Associated Microbiome in Inflammatory Pouch Complications Following Ileal Pouch-Anal Anastomosis
Source: PLoS One. 2013 Sep 24;8(9):e66934. doi: 10.1371/journal.pone.0066934 (PMC3782502; doi:10.1371/journal.pone.0066934)
Supplement: Table S1 — (PDF) [file pone.0066934.s009.pdf]

Table S1: Endoscopic and histological characteristics included in the objective pouchitis score used to define inflammation.

Trait	Severity	Scoring
Erythema	None, mild, severe	0, 2, 3
Friability	None, mild, severe	0, 1, 2
Ulceration	None, mild, severe	0, 2, 3
Polymorphonuclear leukocyte infiltration	None, discrete and patchy (largely confined to surface epithelium), moderate (±) crypt abscesses or cryptitis, extensive (±) crypt abscesses or cryptitis	0, 1, 2, 3
Ulcerations/Erosions	None, mild and superficial, moderate, extensive	0, 1, 2, 3
Total possible score		14
Total score for inflammation		>3
