# Supplementary material for: Characterization of the Gut-Associated Microbiome in Inflammatory Pouch Complications Following Ileal Pouch-Anal Anastomosis
Source: PLoS One. 2013 Sep 24;8(9):e66934. doi: 10.1371/journal.pone.0066934 (PMC3782502; doi:10.1371/journal.pone.0066934)
Supplement: Table S2 — (PDF) [file pone.0066934.s010.pdf]

Table S2: Primers used for pyrosequencing and qPCR experiments.

Primer Target	Primer Names	Forward Primer	Reverse Primer
VI-V3 specific universal primer set	Gray 28F / Gray 519R[20]	GAGTTTGATCNTGGCTCAG	GTNTTACNGCGGCKGCTG
Eubacteria	341-357F / 518-534R[27]	CCTACGGGAGGCAGCAG	ATTACCGCGGCTGCTGG
Bacteroides spp.	Bac303F/ Bfr-Fmrev[48]	GAAGGTCCCCCACATTG	CGCKACTTGGCTGGTTCAG
Roseburia spp. and *E. rectale*	RrecF/ Rrec630mR[48]	GCGGTRCGGCAAGTCTGA	CCTCCGACGCTCTAGTMCGAC
Clostridial cluster IV	Clep866mF / Clept1240mR[48]	TTAACACAATAAGTWATCCACCTGG	ACCTTCCTCCGTTTTGTCAAC
Faecalibacterium prausnitzii	FPR-2F / Fprau645R[48]	GGAGGAAGAAGGTCTTCGG	AATTCCGCCTACCTCTGCACT
Adherent invasive E. coli (AIEC)	EcoliFimH2F / EcoliFimH2R[27]	GCCGGTGGCGCTTTATTTG	TCATCGCTGTTATAGTTGTTGGTCT

Primers listed 5’-3’.
