# Supplementary material for: Characterization of the Gut-Associated Microbiome in Inflammatory Pouch Complications Following Ileal Pouch-Anal Anastomosis
Source: PLoS One. 2013 Sep 24;8(9):e66934. doi: 10.1371/journal.pone.0066934 (PMC3782502; doi:10.1371/journal.pone.0066934)
Supplement: Table S3 — (PDF) [file pone.0066934.s011.pdf]

Table S3: Diversity measures across outcome groups.

	Pouch		Afferent Limb	
	Non-Parametric Shannon Diversity Index	Inverse Simpson Diversity Index	Non-Parametric Shannon Diversity Index	Inverse Simpson Diversity Index
FAP	4.15 (0.13)	25.70 (4.82)	4.11 (0.12)	21.98 (3.39)
NP	3.76 (0.15)	19.94 (3.06)	3.77 (0.11)	17.53 (2.02)
P	3.29 (0.17)	12.14 (1.89)	3.45 (0.21)	15.85 (2.87)
CDL	3.76 (0.11)	20.16 (2.42)	3.72 (0.16)	21.02 (2.42)
P-value	0.006	0.04	0.09	0.35

Values expressed are means and standard error of the mean for pouch and afferent limb samples.
