# Supplementary material for: Characterization of the Gut-Associated Microbiome in Inflammatory Pouch Complications Following Ileal Pouch-Anal Anastomosis
Source: PLoS One. 2013 Sep 24;8(9):e66934. doi: 10.1371/journal.pone.0066934 (PMC3782502; doi:10.1371/journal.pone.0066934)
Supplement: Table S4 — (PDF) [file pone.0066934.s012.pdf]

Table S4: Description of the association between bacterial abundance of the four dominant phyla among pouch and afferent limb samples.

Location	Phylum	P-value				
		FAP vs Pouchitis	FAP vs CDL	No pouchitis vs Pouchitis	No pouchitis vs CDL	4-way analysis
Pouch	Firmicutes	0.64	0.76	0.74	0.51	0.83
	Proteobacteria	0.02	4.0×10^{-3}	0.19	0.03	0.01
	Bacteroidetes	1.0×10^{-4}	1.0×10^{-4}	5.0×10^{-4}	1.0×10^{-3}	1.0×10^{-4}
	Fusobacteria	0.27	0.14	0.79	0.83	0.47
Afferent Limb	Firmicutes	0.66	1.0	0.66	0.90	0.94
	Proteobacteria	0.06	0.01	0.19	0.07	0.06
	Bacteroidetes	1.0×10^{-4}	2.0×10^{-4}	1.0×10^{-3}	0.01	1.0×10^{-4}
	Fusobacteria	0.91	0.91	0.58	0.89	0.96

Nominal p-values are depicted for the overall analysis and for pairwise comparisons. Highlighted rows are those which were significant using the Kruskal-Wallis test and FDR correction for multiple testing. No significant associations were observed in the FAP vs No pouchitis or pouchitis vs. CDL comparisons.
