# Supplementary material for: Characterization of the Gut-Associated Microbiome in Inflammatory Pouch Complications Following Ileal Pouch-Anal Anastomosis
Source: PLoS One. 2013 Sep 24;8(9):e66934. doi: 10.1371/journal.pone.0066934 (PMC3782502; doi:10.1371/journal.pone.0066934)
Supplement: Table S5 — (PDF) [file pone.0066934.s013.pdf]

Supplementary Table 5: Genera achieving a nominal significance level of $P<0.05$ in four-way comparison.

Location	Phylum	Genus	FET P-value	FET P-value (corr)	KWT P-value	KWT P-value (corr)
Pouch	Actinobacteria	*Corynebacterium*	0.02	0.15	0.03	0.16
	Bacteroidetes	*Bacteroides*	$3.6x10^{-6}$	$3.0x10^{-4}$	$5.7x10^{-7}$	$4.7x10^{-5}$
		Parabacteroides	$1.2x10^{-4}$	$4.9x10^{-3}$	$7.8x10^{-4}$	0.02
	Firmicutes	*Moryella*	$2.5x10^{-3}$	0.05	$1.4x10^{-3}$	0.03
		Blautia	$3.0x10^{-3}$	0.05	$2.3x10^{-4}$	$9.5x10^{-3}$
		Dorea	0.02	0.13	0.01	0.09
		Phascolarctobacterium	$9.6x10^{-3}$	0.10	0.02	0.15
		IS *Erysipelotrichaceae*	0.02	0.14	0.03	0.16
		Dialister	0.02	0.13	0.02	0.15
		Uncultured *Lachnospiraceae*	0.04	0.20	$6.9x10^{-3}$	0.07
		Uncultured *Ruminococcaceae*	0.04	0.20	$7.1\ x10^{-3}$	0.07
		Subdoligranulum	0.01	0.13	0.03	0.16
		Uncultured Family XIII IS	$8.5\ x10^{-3}$	0.10	$7.7x10^{-3}$	0.07
		Roseburia	0.07	0.31	0.04	0.17
	Proteobacteria	*Sutterella*	$2.5x10^{-3}$	0.05	$2.4x10^{-3}$	0.04
		Rhodobacter	0.01	0.09	0.03	0.16
		Haemophilus	0.13	0.39	$5.9x10^{-3}$	0.12
Afferent Limb	Actinobacteria	Uncultured *Coriobacteriaceae*	0.03	0.17	0.03	0.20
		Collinsella	0.02	0.16	0.02	0.19
	Bacteroidetes	*Bacteroides*	$6.9x10^{-6}$	$2.9x10^{-4}$	$4.2x10^{-6}$	$3.4x10^{-4}$
		Parabacteroides	$2.2x10^{-3}$	0.03	$8.8\ x10^{-3}$	0.10
	Firmicutes	*Moryella*	0.03	0.17	0.03	0.20
		Blautia	$8.2x10^{-7}$	$6.8x10^{-5}$	$1.1x10^{-4}$	$4.7x10^{-3}$
		Dorea	$1.0x10^{-3}$	0.03	$4.2x10^{-3}$	0.07
		Anaerococcus	$2.8x10^{-3}$	0.05	$8.4x10^{-3}$	0.11
		IS *Erysipelotrichaceae*	$4.5x10^{-3}$	0.05	$4.0x10^{-3}$	0.07
		Roseburia	0.02	0.15	0.08	0.30
		Uncultured Family XIII IS	0.01	0.13	0.03	0.19
		Granulicatella	0.07	0.31	0.03	0.19
		Dialister	0.05	0.23	0.05	0.21
		Lactobacillus	0.03	0.17	0.01	0.15
		Mogibacterium	0.04	0.20	0.05	0.21
		Phascolarctobacterium	0.02	0.16	0.03	0.19
	Proteobacteria	*Sutterella*	$1.7x10^{-3}$	0.03	$8.5x10^{-4}$	0.02
		Rhodobacter	0.03	0.17	0.05	0.21
		Haemophilus	0.12	0.37	0.05	0.21

FET=Fisher's exact test (dichotomous results); KWT=Kruskal-Wallis Test (continuous results). Highlighted rows are those which remained significant in either analysis after FDR correction for multiple testing.
